# Subcortical brain structures among maltreated youth with high and low externalizing problems

**DOI:** 10.1007/s00787-025-02927-y

**Published:** 2025-12-08

**Authors:** Charlotte C. Schulz, Lara M. C. Puhlmann, Lisa Folkens, Kai von Klitzing, Lorenz Deserno, Pascal Vrtička, Lars O. White

**Affiliations:** 1 Department of Child and Adolescent Psychiatry, Psychotherapy, and Psychosomatics, University of Leipzig, Leipzig, Germany; 2 Max Planck Institute for Human Cognitive and Brain Sciences, Leipzig, Germany; 3 Leibniz Institute for Resilience Research, Mainz, Germany; 4 Faculty of Psychology, Clinical Psychology and Behavioral Neuroscience, Technische Universität Dresden, Dresden, Germany; 5 Department of Child and Adolescent Psychiatry, Psychosomatics and Psychotherapy, University of Würzburg, Centre of Mental Health, Würzburg, Germany; 6 Department of Psychiatry and Psychotherapy, Technische Universität Dresden, Dresden, Germany; 7 Department of Psychology, Centre for Brain Science, University of Essex, Colchester, United Kingdom; 8 Department of Clinical Child and Adolescent Psychology and Psychotherapy, University of Bremen, Bremen, Germany

**Keywords:** Brain structure, Abuse, Maltreatment, Psychopathology, Adolescence

## Abstract

Alterations in brain structure following adversity show inconsistencies. Jointly considering maltreatment exposure alongside externalizing psychopathology may therefore prove helpful. However, few prospective studies currently follow up maltreated youth with high or low externalizing problems using neuroimaging assessments. A structural magnetic resonance imaging study was conducted using T1-weighted scans from 98 youth (*M*_Age_ = 14.76, *SD*_Age_ = 1.96; 51% girls) comparing participants with low (*n* = 29) and high (*n* = 29) maltreatment exposure to carefully matched non-maltreated controls (*n* = 40). Comprehensive assessments of maltreatment history and psychopathology were ascertained across two waves, applying the Maltreatment Classification System alongside caregiver- and teacher-reported externalizing problems. Neuroimaging was conducted at the second wave. We found dose-dependent amygdala volume reductions following maltreatment and particularly abuse exposure, primarily driven by the basolateral subregion. Interaction analyses revealed lower amygdalar and hippocampal volumes in youth exposed to more vs. less extensive maltreatment, but merely among those with elevated externalizing problems. These findings highlight the value of the ecophenotype model for elucidating neural correlates of maltreatment and its sequelae.

**Supplementary Information:**

The online version contains supplementary material available at 10.1007/s00787-025-02927-y.

## Introduction

Child maltreatment ranks among the most deleterious adversities for mental health [1, 2]. A common developmental pathway following maltreatment, particularly threat exposure (including abuse and domestic violence), involves higher risk of externalizing psychopathology across the lifespan [3, 4]. This pattern potentially contributes to the intergenerational transmission of maltreatment [5]. However, the neurobiological substrates of this “cycle of violence” remain incompletely understood. While structural alterations in threat-processing brain regions such as the amygdala and hippocampus have been implicated in maltreatment exposure, particularly involving abuse [6–8], and externalizing psychopathology [9, 10], there have been few attempts to integrate these separate lines of research [11–13]. The present study seeks to fill this gap by examining the interplay between maltreatment and externalizing problems in relation to neurostructural substrates in youth.

The hippocampus and amygdala figure prominently as structural correlates of child maltreatment. While a maltreatment history coincides with reduced hippocampal volume in adults [14], findings in youth reveal inconsistencies [8, 15]. Gradual volume decreases across development detected by longitudinal studies [16, 17] may account for mixed results in cross-sectional research [18, 19]. Conversely, neither in youth nor in adults exposed to child maltreatment, a consistent pattern of effects on amygdala volume has emerged [6, 14, 20, 21]. Nonetheless, at least two factors might help resolve this heterogeneity: First, a well-established measure of maltreatment pervasiveness may clarify the picture, as greater exposure more reliably coincides with reduced amygdala volume [18, 22]. Second, studies that zero in on circumscribed subtypes or dimensions yield more consistent findings. Thus, in keeping with the “dimensional model of adversity and psychopathology” (DMAP; [23]), neuroimaging studies in youth exposed to adversity [7] indicate volume reductions in the hippocampus, amygdala, and medial prefrontal cortex (PFC) related to threat (including abuse), but not deprivation (including neglect). However, previous work typically was not designed to directly assess specific and relative effects of differential adversity exposures, often neglecting the common co-occurrence of threat and deprivation [7, 24]. While recent research has begun to address this issue, it yields incongruent results, with lower amygdala volume linked to threat in one study [19], but not the other [25].

In a similar vein, when examining neurostructural correlates of mental health conditions recent neuroimaging research emphasizes the moderating influence of adversity [26, 27]. Indeed, structural correlates of these conditions could be partly attributable to the impact of prominent environmental risks for the same conditions, including maltreatment [28]. Specifically for externalizing problems, meta-analytic and large-scale data support decreased amygdalar and hippocampal volumes as the most reliable findings [9, 10]. However, for subtypes of externalizing problems, research has been less forthcoming [9]. Inasmuch as maltreatment exerts a greater influence on certain trajectories and/or subtypes of externalizing problems (e.g., early onset, life-course persistent) over others (e.g., limited prosocial emotions or anxiety; [29, 30]), the question arises whether neurobiological correlates of externalizing problems may vary as a function of a maltreatment history [27]. While two recent studies address this gap [11, 12], this work was hitherto merely designed to consider maltreatment retrospectively and in terms of a dichotomous phenomenon (presence/absence), which does not do justice to its heterogeneity or the dimensional perspective outlined above [31]. Moreover, as prior work selected children based on conduct disorder (CD) diagnoses followed by retrospective assessments of maltreatment, this begs the question whether such interactions are also observable in prospective designs that follow up samples exposed to maltreatment with varying levels of externalizing outcomes.

Consequently, we expressly designed our study to ensure sufficient levels of maltreatment exposure, while simultaneously enabling comparisons with matched non-maltreated controls. Maltreatment was assessed prospectively at two time points to capture chronicity, severity, subtype, and timing. As retrospective and prospective assessments identify partly different individuals [32], we aimed to extend previous research by examining associations of prospectively reported maltreatment and externalizing problems, as well as their interplay, with hippocampal and amygdalar structure in adolescents with varying maltreatment exposure and matched controls. We hypothesized that maltreatment-exposed adolescents would show reduced amygdalar and hippocampal volumes compared to controls, with the strongest reductions in the high-exposure group (vs. both no and low exposure). We further predicted that maltreatment pervasiveness, particularly abuse exposure-level, would relate negatively to subcortical volumes. Finally, we expected externalizing problems to be associated with reduced amygdalar and hippocampal volumes, moderating the link between maltreatment exposure and subcortical volumes.

## Methods

### Sample

We selected 12-to-17-year-olds with maltreatment histories (*n* = 63) and matched non-maltreated controls (*n* = 47) from a pre-existing large-scale sample of maltreated and non-maltreated youth, originally recruited from child protection services (CPS), child and adolescent psychiatric services (CAPS), and the community in the AMIS Project [33, 34] (*N* = 851; see Figure A1 for study timeline). Almost half of the present maltreated sample (*n* = 27; 46.6%) reported previous CPS contact. Exclusion criteria for the neuroimaging subsample have been reported elsewhere [35].

Of the 110 initially recruited participants, 12 were excluded due to magnetic resonance imaging (MRI) contraindications (e.g., piercings, anxiety during mock scan; *n* = 3), incidental findings (*n* = 3), substance abuse (*n* = 1), or excessive movement (*n* = 5; for details, see section **Image pre-processing**). The final sample included 98 adolescents (51% girls; age 14.76 ± 1.96 years), grouped as a function of maltreatment exposure-level into no (*n* = 40) vs. low (*n* = 29) vs. high (*n* = 29) maltreatment exposure groups, and matched for age, gender, IQ, and handedness (see Table B1).

The Institutional Review Board of the University of Leipzig provided ethical approval for the study. We obtained informed written and oral consent (caregivers) and oral assent (adolescents) from all participants. Families received monetary compensation for participation.

### Maltreatment characteristics

We applied the Maltreatment Classification System (MCS; [36]) to score participants’ maltreatment histories using the 30–120 min Maternal Maltreatment Classification Interview (MMCI; [37]) as well as their CPS records, if accessible (*n* = 9). The MCS is a rigorous well-validated, reliable coding scheme to evaluate child maltreatment, with stringent definitions based on examples derived from CPS files [38]. We computed mean chronicity (percentage of affected developmental periods), mean maximum severity (1–5), and subtype number for overall maltreatment (1–6), abuse (1–3: sexual, physical, emotional), and neglect (1–3: moral-legal/educational, physical, emotional). For each maltreatment subtype, we further assessed maltreatment onset and recency. Binary indicators of onset (infancy/toddlerhood vs. later developmental periods) and recency (last incident in current vs. previous developmental periods) were derived for overall maltreatment, abuse, and neglect. As maltreatment was first assessed on average 5.50 years (*SD* = 0.52) before neuroimaging (i.e., Wave 1), a second MMCI with caregivers at Wave 2 (i.e., neuroimaging wave) captured interim incidents. All available data across both waves were integrated. Participants were classified as maltreated if any reported incident fulfilled the criteria stipulated by the MCS at either wave. Confirmatory factor analyses (MPLUS, v7.4; [39]) in the full AMIS sample yielded aggregate factor scores for the extent of overall maltreatment as well as abuse and neglect exposure based on their respective mean chronicities, mean maximum severities, and subtype numbers across both waves (see **Appendix A** for more detailed information). Thus, these three variables serve as indices of overall maltreatment, abuse, and neglect pervasiveness on a continuous scale with higher scores reflecting greater severity, chronicity, and/or number of maltreatment subtypes. We then divided maltreatment-exposed participants (*n* = 58) into two subgroups using the median of the dimensional indicator, yielding 29 adolescents with low (41.38% females, age 14.66 ± 1.95 years) and 29 with high (48.28% females, age 15.02 ± 2.13 years) maltreatment exposure.

### Psychopathological symptoms

Adolescents’ internalizing and externalizing problems were assessed with the Strengths and Difficulties Questionnaire (SDQ; [40]) and the Child Behavior Checklist (CBCL; [41]) at both waves. At Wave 1, mothers, fathers, and teachers reported on participants’ emotional symptoms and conduct problems in the SDQ as indicators for internalizing and externalizing problems, respectively. Additionally, we obtained CBCL caregiver-reports on externalizing and internalizing symptoms at both waves. Participants who scored above the population-derived borderline or clinical cut-offs for either internalizing or externalizing symptoms in any of these assessments were classified into the groups of high internalizing (*n* = 61) and externalizing problems (*n* = 31), respectively (see** Appendix A** for detailed information on CBCL, SDQ, and the classification approach).

### Covariates

To account for potential confounders and characterize the sample, we assessed adolescents’ pubertal status, IQ, and maternal education as a proxy for socioeconomic status (SES). We also screened adolescents for features of fetal alcohol syndrome (FAS). Details on how these covariates were operationalized are provided in **Appendix A**.

### MRI data acquisition

At Wave 2, structural brain scans were acquired on a 3 T Siemens Skyra scanner with a 32-channel head coil. A T1-weighted Magnetization Prepared Rapid Gradient Echo (MPRAGE) sequence (TR = 2300ms, TE = 2.98ms, flip angle = 9°, FoV = 256 mm, voxel size: 1 × 1 × 1 mm, 176 slices; [42, 43]) was used. Foam cushions were placed between participants’ heads and the coil to reduce head motion and increase comfort during the scanning procedure (see **Appendix A** for details).

### Data analysis

#### Image pre-processing

First, two trained research assistants independently checked all T1-weighted images for structural abnormalities, movement artifacts, and other image distortions rating the image quality on a scale from 1 (very high) to 5 (very low). Cases with an image quality of 4 or 5 were excluded, while for cases with medium image quality (i.e., 3) a joint decision was made. Subsequently, Freesurfer, v6.0 (https://surfer.nmr.mgh.harvard.edu*)* was employed for image pre-processing applying intensity normalization and skull stripping procedures to the T1-weighted brain image. Then, the software computes brain volume and performs surface-based reconstruction to measure cortical thickness. Reconstruction of cortical and subcortical brain regions was manually corrected by two trained graduate students (see **Appendix A**).

For our ROI analyses, we extracted subcortical volume measurements (in mm^3^) and estimated total intracranial volume (ICV) employing Freesurfer, v6.0 [44]. Additionally, estimates for total cortical gray matter (GM) and cerebral white matter (WM) volume were retrieved to assess maltreatment effects on global cortical GM and cerebral WM (see **Appendix A** for methods and **Appendix B** for results). To obtain volumetric measurements of hippocampal subfields and amygdalar nuclei for exploratory analyses, we further processed the images using an automated segmentation pipeline of Freesurfer, v7.1 [45, 46] which only became available during the course of the project and after manual corrections had already been conducted. We calculated mean subcortical volumes across both hemispheres as target outcomes, as our hypotheses make no distinction between them. Correlations between left and right amygdalar and hippocampal volumes (including subregions) are reported in Table B2 and Table B3. For correlations between all maltreatment and mental health indicators as well as ROI volume estimates, refer to Table B4 and Table B5.

#### Region-of-interest analyses

We first tested for maltreatment effects (no vs. low vs. high exposure) on amygdala and hippocampal volumes. Next, we assessed effects of high vs. low externalizing problems and dimensional associations of parent-reported externalizing symptoms at Wave 2 with subcortical ROIs. Third, we examined the interaction effect between maltreatment exposure and externalizing problems. To increase power and facilitate interpretation of the results, analyses were repeated using a regrouped binary maltreatment variable (i.e., combined no and low vs. high exposure-level). Significant interactions were followed up with moderation analyses of dimensional parent-reported symptoms at Wave 2 using the PROCESS macro, v4.1 [47] to test their dose-dependency and relevance for concurrent symptomatology. To assess specificity to externalizing problems, main and interaction effects were also tested for internalizing problems. Within individuals with the respective maltreatment exposure, dose-dependent effects of overall maltreatment (*n* = 58) and of abuse and neglect (*n* = 47 each) on amygdalar and hippocampal volumes were examined. Unadjusted dimensional effects of abuse and neglect were tested before modeling both types of effects, while controlling for one another. Analyses were conducted with and without covariates (ICV, age, gender, SES, as well as maltreatment onset and recency) and, for mental health effects, the other psychopathology type. Results are reported with covariate adjustment unless noted otherwise (see **Appendix B** for unadjusted results). In sensitivity analyses, we assessed lateralization for significant effects on total volume indicators (see **Appendix B** for results). All analyses controlled for multiple comparisons (*q* < 0.025) and adjusted for data distributions (i.e., normality vs. non-normality, homoscedasticity vs. heteroscedasticity).

Supplemental analyses tested whether maltreatment effects on total amygdalar and hippocampal volume were driven by alterations to stress-sensitive hippocampal subfields (i.e., cornu ammonis (CA) subfields 2/3 and CA4/dentate gyrus, DG) and amygdalar subregions (i.e., the basolateral complex, BLA), with multiple-comparison thresholds of *q* < 0.025 for two amygdala segments (i.e., BLA and centrocorticomedial complex, CMA) and *q* < 0.0125 for four hippocampal subfields (i.e., subiculum, CA1, CA3 (including CA2), and CA4/DG; see **Appendix A** for analysis details including model equations).

## Results

### Main effects of maltreatment exposure, internalizing and externalizing problems

**Fig. 1:**
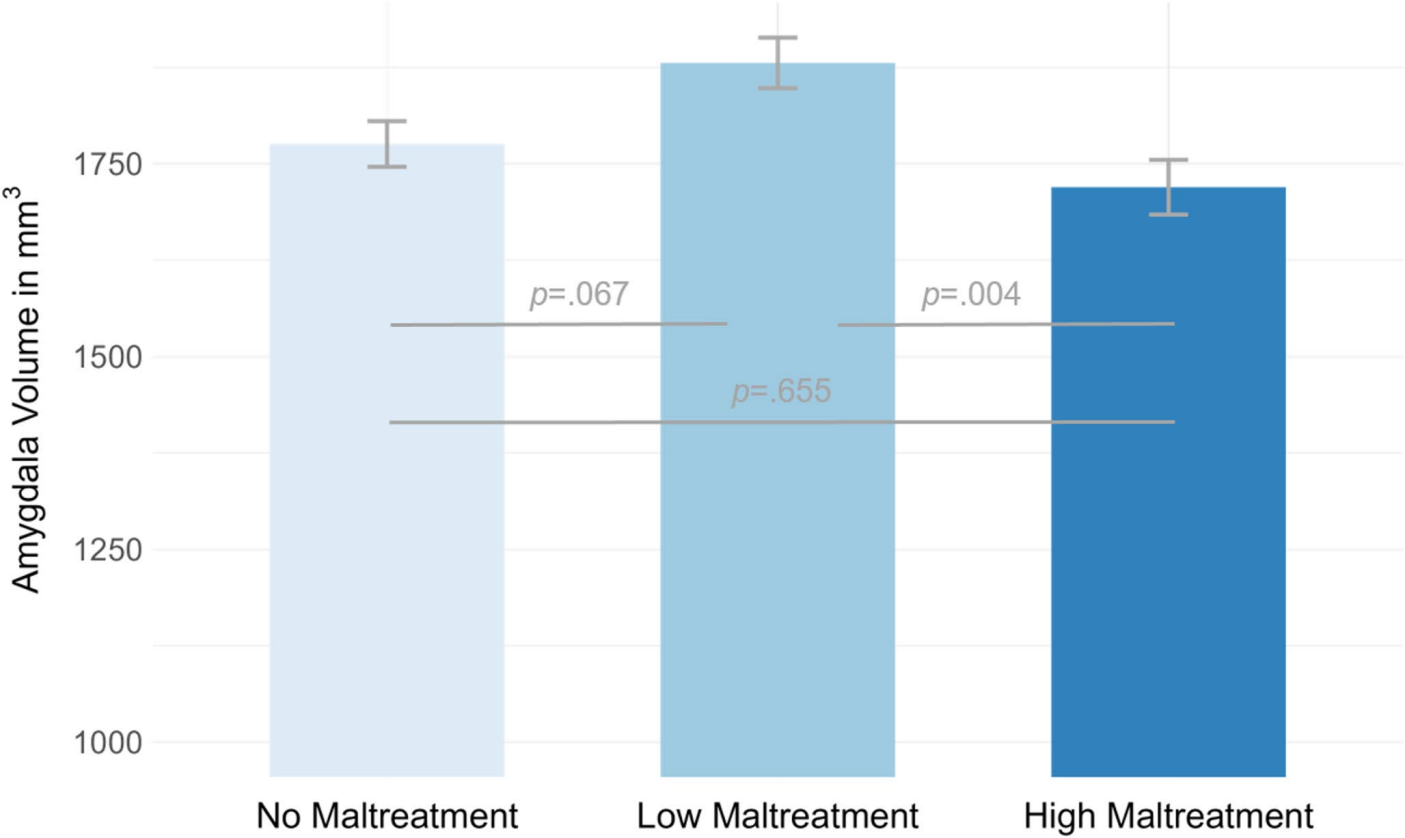
Mean amygdala volumes and respective standard errors for non-maltreated (*n* = 40) vs. low (*n* = 29) vs. high maltreatment-exposed participants (*n* = 29)

While no main effect of maltreatment exposure emerged for bilateral hippocampal volume (*F*(2, 91) = 0.46, *p* = .633), bilateral amygdala volume differed between the three exposure subgroups (*F*(2, 91) = 4.24, *p* = .017, η_p_^2^ = .09; see Fig. 1 and Table B6). This effect further survived controlling for maltreatment recency and onset (*p*s ≤ .021). Bonferroni-corrected pairwise post-hoc comparisons showed amygdala volumes were higher in low than in high maltreatment-exposed participants and marginally higher than in controls (see Fig. 1 and Table B7).

We further found a main effect of internalizing problems on hippocampal volume (*F*(1, 91) = 6.85, *p* = .010, η_p_^2^ = .07), pointing to reduced volume in participants with high (*M* = 4120.98, *SD* = 382.52) vs. low internalizing problems (*M* = 4240.11, *SD* = 327.24). Contrastingly, internalizing problems did not exert a main effect on amygdala volume (*F*(1, 91) = 0.19, *p* = .663). Further, no main effects of externalizing problems on amygdalar or hippocampal volumes emerged (*p*s ≥ .408). Similarly, concurrent internalizing symptoms were negatively associated with hippocampus volume at trend-level (β = −.19, *p* = .060, *R*^2^ = .53), whereas dimensional psychopathology indicators were not related to amygdala volume (*p*s ≥ .181).

### Interaction effects of maltreatment exposure with internalizing and externalizing problems

As hypothesized, maltreatment exposure interacted with externalizing problems to explain variance in total amygdalar (*F*(2, 87) = 5.58, *p* = .005, η_p_^2^ = .11) and hippocampal volumes (*F*(2, 87) = 6.91, *p* = .002, η_p_^2^ = .14; see Table B8). These interaction effects also survived controlling for maltreatment recency and onset (*p*s ≤ .006). In contrast, there were no interaction effects with internalizing problems for bilateral amygdalar and hippocampal volumes (*p*s ≥ .263).

**Fig. 2:**
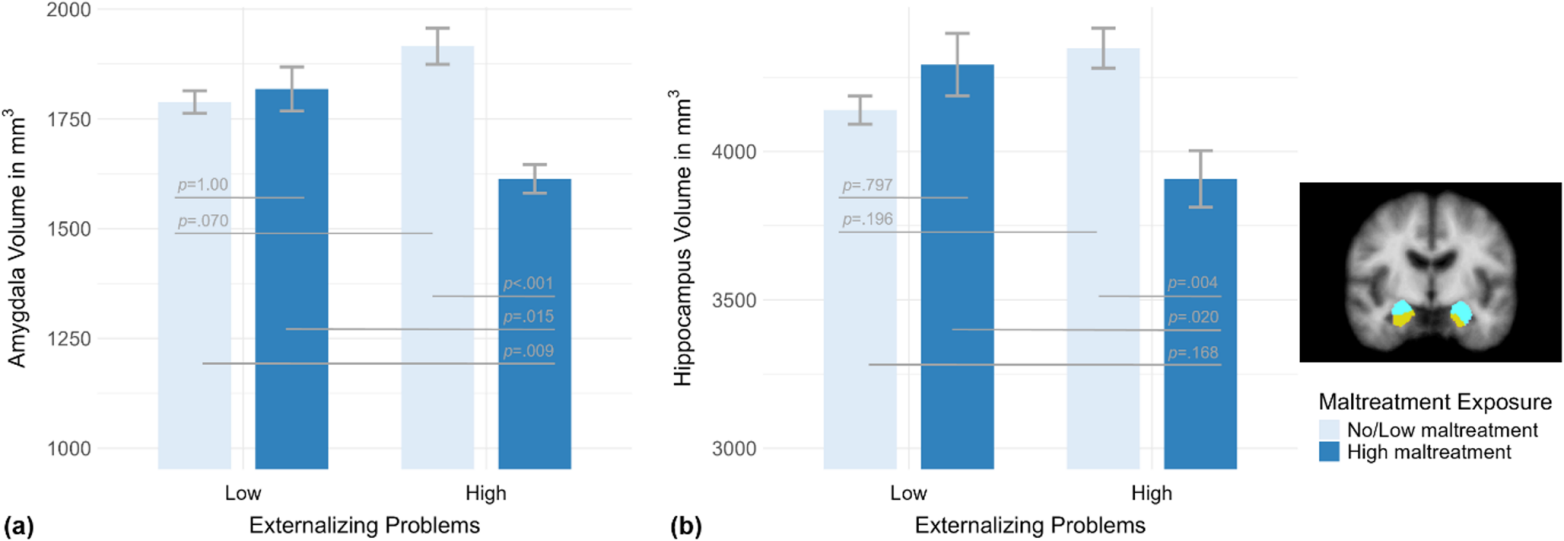
Mean (**a**) amygdala and (**b**) hippocampus volumes and respective standard errors for adolescents with combined no/low (light blue) vs. high maltreatment exposure (dark blue) within the subgroups of participants with low vs. high externalizing problems

Due to small group sizes in the high externalizing subgroups with no (*n* = 6) and low maltreatment exposure (*n* = 11) and the absence of subcortical volume differences^1^, we combined these groups to maximize power. Applying the re-grouped, binary maltreatment variable (i.e., no and low vs. high exposure-level), the interaction effect between maltreatment exposure and externalizing problems remained significant for amygdalar (*F*(1, 89) = 12.11, *p* < .001, η_p_^2^ = .12) and hippocampal volumes (*F*(1, 89) = 12.79, *p* < .001, η_p_^2^ = .13; see Fig. 2 and Table B9).^2^ The interaction effects survived correcting for maltreatment recency and onset (*p*s ≤ .001). Refer to Fig. 2 and Table B10 for Bonferroni-corrected post-hoc comparisons.

**Fig. 3:**
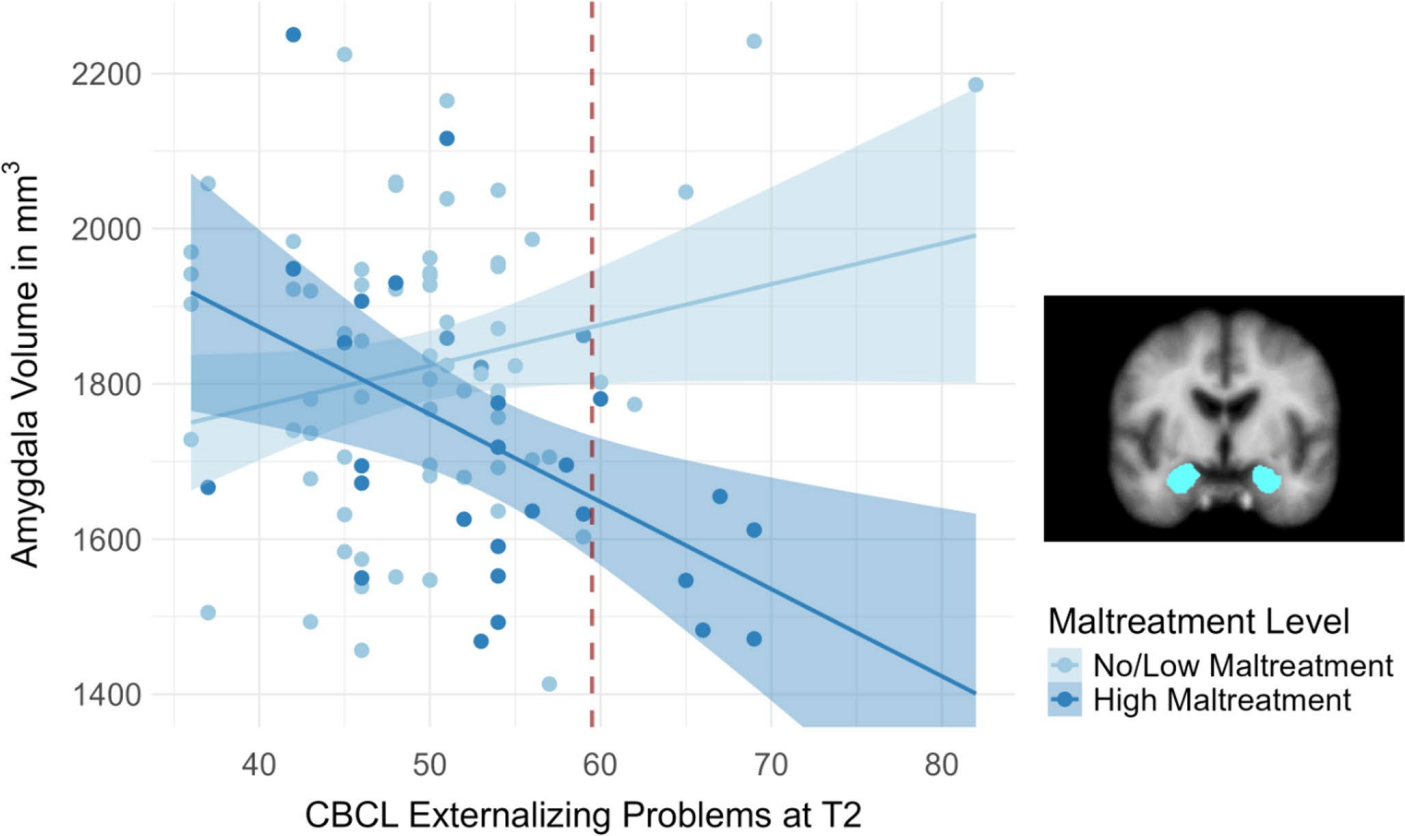
Association between concurrent externalizing problems (t-values) at T2 (i.e., neuroimaging wave) assessed with the Child Behavior Checklist (CBCL; x-axis; red dashed line displays at-risk cut-off) and amygdala volume (y-axis) for participants with combined no and low maltreatment (*r* = .22, *p* = .068; *n* = 69; light blue) compared to those with high maltreatment exposure-level (*r *= -.49, *p* = .007; *n* = 29; dark blue)

In line with the categorical interaction effect, dimensional caregiver-reported externalizing problems at Wave 2 moderated the effect of maltreatment exposure on amygdala volume (β = −.24, *p* = .017, *R*^2^ = .52; see Fig. 3 and Table B11). This moderation effect further survived adjustment for maltreatment recency and onset (*p*s ≤ .022). For bilateral hippocampal volume, however, the dimensional moderation effect of concurrent externalizing problems (β = −.32, *p* = .011, *R*^2^ = .08) did not survive covariate adjustment (β = −.15, *p* = .117).

### Within-group effects of maltreatment dimensions

**Fig. 4:**
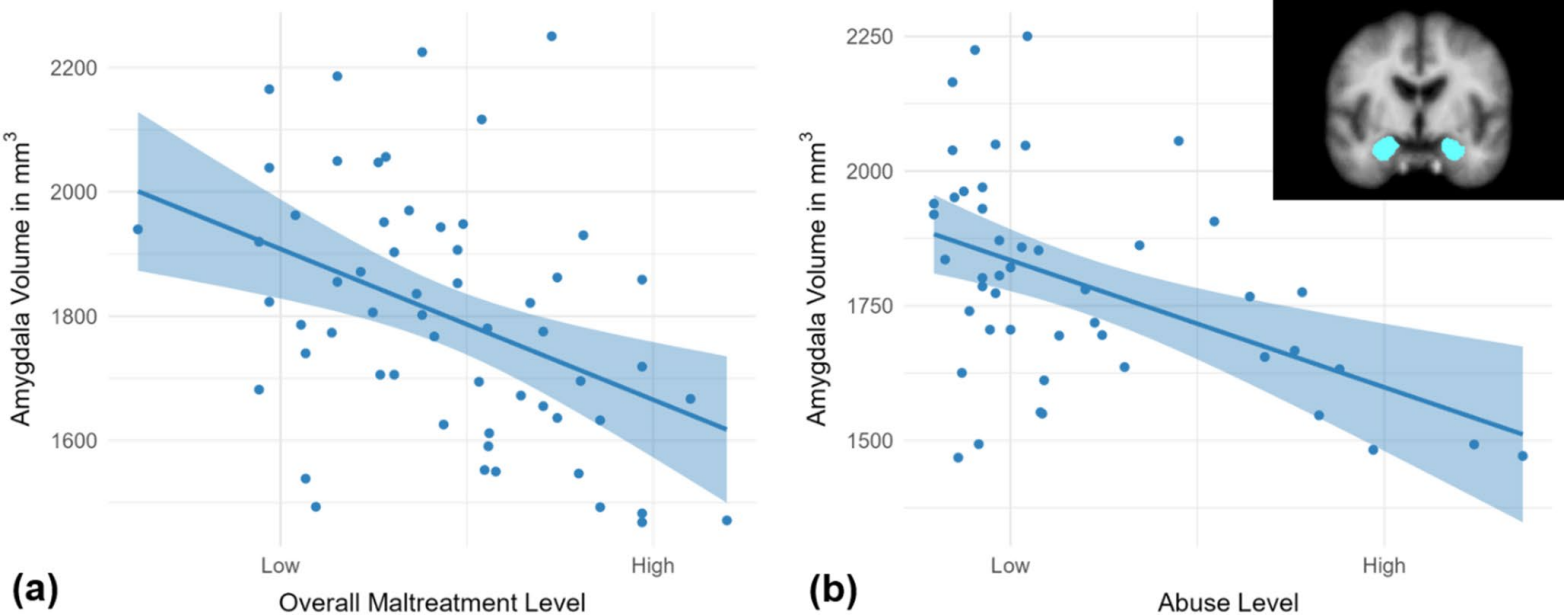
Negative associations of (**a**) overall maltreatment and (**b**) abuse exposure-level (factor scores, x-axis) with amygdala volume (y-axis) within participants exposed to maltreatment (*r *= -.41, *p* = .001; *n* = 58) and abuse (*r *= -.48, *p* < .001; *n* = 47)

In line with the main effect of maltreatment exposure across the whole sample, dimensional within-group analyses showed a negative dose-response relationship between overall maltreatment exposure-level and amygdala volume (β = −.32, *p* = .006, *R*^2^ = .53; see Fig. 4a and Table B12). This association further survived controlling for maltreatment recency and onset (*p*s ≤ .014). Contrastingly, we did not find a significant association of overall maltreatment exposure-level and hippocampal volume (β = −.11, *p* = .305).

Within the subgroup of participants with a history of abuse (*n* = 47), abuse exposure-level was negatively related to total amygdala volume (β = −.31, *p* = .017, *R*^2^ = .53 adjusted for neglect; see Fig. 4b and Table B12). This association remained significant when controlling for abuse recency and onset (*p*s ≤ .023). For the neglect dimension, however, no significant association with total amygdala volume emerged (β = −.04, *p* = .795 adjusted for abuse; *n* = 47). Abuse and neglect exposure-levels were not significantly related to hippocampal volume (*p*s ≥ .777).

### Supplemental analyses

Most importantly, while no maltreatment main effects on amygdalar or hippocampal subregion volumes survived covariate adjustment (*p*s ≥ .056), maltreatment interacted with externalizing problems to predict BLA, CA1, CA3, CA4/DG, and subiculum volumes (*p*s ≤ .011; recoded, binary maltreatment: *p*s ≤ .004). In maltreatment-exposed youth, overall maltreatment was negatively associated with BLA volume (*p* = .008), with abuse showing a trend-level association (*p* = .034; *q* < .025). No significant associations with abuse or neglect emerged for other amygdalar (*p*s ≥ .124) or hippocampal (*p*s ≥ .175) subregions (see **Appendix B** for detailed results).

## Discussion

Child maltreatment incurs a substantial burden on mental health, including externalizing problems as one of the most common outcomes [3, 4]. Notably, partly overlapping subcortical alterations have been reported in youth with a history of child maltreatment and those with externalizing problems [7, 9]. This study integrates previous research on brain structure alterations in relation to both child maltreatment and externalizing problems by considering their joint neurodevelopmental correlates. Complementing previous research, our study incorporated fine-grained, multi-source maltreatment assessments enabling the structured coding of maltreatment type, severity, chronicity, and timing across developmental periods. Results showed dose-dependent amygdala volume reductions with increasing maltreatment, and particularly abuse exposure-level, primarily driven by volume differences in the basolateral amygdala. Compared to non-maltreated and high-exposure groups, participants with low maltreatment exposure showed higher amygdala volume, with no difference between high and non-maltreated groups. Strikingly, however, interaction analyses revealed remarkable decreases in amygdalar and hippocampal volumes in youth exposed to extensive maltreatment, if they additionally manifested elevated externalizing problems. Conversely, internalizing problems showed a main effect for reduced total hippocampus and CA3 subfield volume, but no interaction with maltreatment.

Our data show an inverse relationship between the degree of maltreatment exposure and amygdala volume. When testing this association for abuse and neglect separately, only abuse exposure-level specifically predicted amygdala volume reductions – even after controlling for co-occurring neglect as well as maltreatment recency and onset. This finding aligns with recent research [19] reporting threat-specific reductions in amygdala volume, especially in children up to 13 years, with comparable effect sizes (i.e., small to medium). However, Machlin et al. [25] did not replicate an abuse-specific effect in a longitudinal study linking adversity measured in preschool to brain structure in 5–10 year-olds. As both studies examined children of similar age, but differed in timing of adversity assessment, recency and chronicity may be critical for predicting amygdala volume. Our study bridges these disparate findings by highlighting the impact of chronic maltreatment and specifically abuse exposure, while controlling for maltreatment recency and onset. Thus, while focusing on a narrower area of adversity (i.e., maltreatment), our data support the DMAP [23], which posits that threat experiences (including abuse) specifically affect brain regions involved in emotional learning, such as the amygdala. At the same time, given that our study focused on subcortical regions, it was not designed to assess the effects of deprivation (or neglect) which the DMAP primarily hypothesizes to affect cortical regions.

Group differences between non-maltreated, low, and high maltreatment-exposed participants suggest an inverted U-shaped association between maltreatment and amygdala volume. A similar non-linear pattern has been proposed by Hanson and colleagues [18, 20], with cross-sectional and longitudinal studies indicating initial amygdala growth followed by reductions under severe or chronic adversity [22, 48]. Our findings support this pattern, but replication using dimensional, non-linear analyses is needed. As dimensional maltreatment data were only available for the exposed group, we could not test for a non-linear, dimensional association across the full sample.

In our study, differences in hippocampal and amygdalar volumes were best accounted for by an interaction between maltreatment exposure and externalizing problems, but not internalizing problems. Youth with high externalizing problems and extensive maltreatment thus showed reduced amygdalar and hippocampal volumes compared to those with low externalizing problems and either no/low or high maltreatment exposure. Moreover, in highly maltreatment-exposed adolescents, amygdala volume decreased with increasing externalizing problems. While these findings align with our hypotheses, based on meta-analytic and large-scale imaging research on externalizing disorders [9, 10], they appear somewhat inconsistent with recent research on youth with CD. Notably, research points to both increased amygdala volume [11] and no group differences in subcortical volume [12], when comparing youth with CD with and without a history of child maltreatment.

Several considerations could explain these inconsistencies. First, part of these inconsistencies may be attributable to the time-dependency of the stress-response related to initial neuroplasticity and growth followed by neurotoxic processes and volumetric decrease [15]. Maltreatment may initially promote amygdala growth, but increased stress sensitivity could subsequently lead to volume reduction [20]. Therefore, variations in the samples’ maltreatment chronicity, onset, and recency might contribute to divergent findings. Second, differences in sample composition due to varying recruitment strategies (e.g., age, gender) may play a role. More specifically, complementing previous work [11, 12], we assessed child maltreatment prior to psychopathology and went well beyond mere binary assessments of absence vs. presence of maltreatment [31], also considering chronicity and timing of exposure. This allowed us to target and meticulously assess varying maltreatment exposure-levels, including severe cases documented by our multiple MCS assessments and CPS-involvement. Our research indicates that externalizing problems are primarily associated with reduced subcortical volume at high levels of maltreatment. Thus, our study highlights the importance of considering maltreatment pervasiveness and timing rather than merely its presence or absence, thereby adding to the ongoing debate in this research field [31].

Crucially, our findings also indicate that the interaction effect on subcortical volumes is specific to externalizing symptoms, as no corresponding effect emerged for internalizing problems. This aligns with research in children reporting no interaction between maltreatment and anxiety disorders regarding alterations in brain structure [25]. Conversely, however, studies in adults with affective disorders report subcortical alterations as a function of retrospective and largely self-reported maltreatment [49]. Consequently, results may vary by age group and maltreatment assessment method.

Although subcortical volume alterations might not be explained by an interaction between maltreatment and internalizing problems, we found reduced total hippocampal and CA3 subfield volumes in adolescents with high internalizing problems. This aligns with prior findings of negative associations between internalizing symptoms and total hippocampal and subfield volumes [50, 51], suggesting a vulnerability mechanism. By contrast, adversity-related functional connectivity changes in the cingulo-opercular network (including the hippocampus) predicting lower internalizing symptoms may represent an adaptive response [52].

We also observed specific structural alterations in amygdalar and hippocampal subregions linked to maltreatment and externalizing problems. Consistent with our hypotheses and previous research [53, 54], amygdalar volume reductions related to maltreatment were driven by alterations in the stress-sensitive BLA. While previous studies report effect sizes in the small-to-large range [53, 54], we detected medium effects. Beyond this main effect, ours is the first study to show that the moderating influence of externalizing problems for the effect of maltreatment on total amygdala volume may be driven by differences in BLA rather than CMA structure. Animal studies highlight the BLA’s role in fear conditioning [55] and anxiety-like behaviors following stress [56]. The BLA’s stress susceptibility can further be explained by its high glucocorticoid receptor density [57]. Thus, our study supports and extends prior research in youths and young adults showing amygdalar nuclei alterations following adversity [53, 54].

By contrast, we found no main effects of maltreatment on the hippocampus and its subfields, contradicting previous reports of reductions in total hippocampus and especially CA2/3 and CA4/DG subfield volumes in maltreatment-exposed youth [18, 58, 59]. Instead, the interplay between maltreatment exposure and externalizing problems explained variance in all examined hippocampal subfields (i.e., CA1, CA2/3, CA4/DG, and subiculum), supporting global as opposed to subfield-specific alterations. Thus, rather than exerting individual effects, maltreatment exposure and externalizing problems appear to jointly give rise to hippocampal alterations. This pattern dovetails with the assumption that adversity has a delayed effect on the hippocampus development [16, 17], possibly due to the onset of psychopathology following maltreatment exposure.

### Limitations

Several limitations warrant consideration. First, unlike prior work [11, 12], we focused on fine-grained maltreatment assessments instead of CD diagnoses. Thereby, our findings complement existing research highlighting the need to distinguish externalizing from non-externalizing pathways within maltreated populations [60]. Underlying neural mechanisms may emerge subclinically as formative risk factors. Second, while 47% of the maltreated sample divulged CPS contact – in line with high levels of risk and a considerable degree of trust in the interviewer - CPS records were available for only 15.5% of the maltreatment-exposed participants. For most participants, maltreatment assessments therefore relied solely on parent reports, which may be biased. However, restricting analyses to cases with CPS data would reduce variability needed to test dimensional adversity models like the DMAP. Third, given our focus on maltreatment sequelae, we controlled for other adversities (e.g., SES) through group matching or covariates. Thereby, we also accounted for related factors (e.g., neighborhood violence, cognitive deprivation), which increases specificity but may reduce comparability with studies on broader adversity constructs [19, 25]. Fourth, small effect sizes in neuroimaging research raise concerns about statistical power [61], which could explain our null findings for main effects of externalizing symptoms [9] and interaction effects with internalizing problems though our sample notably still exceeds the size typical in this field [62]. Nevertheless, given the high variance in internalizing symptoms (62.25% above cut-off), the null finding may reflect a true effect rather than a power issue. Fifth, using different Freesurfer versions for preprocessing and manual correction (v6.0) versus subregion segmentation (v7.1) may limit reliability and generalizability of our exploratory subregion analyses. This was necessary, however, as v6.0 did not fully support amygdala subregion segmentation. Although no systematic evaluation of this issue exists, prior research suggests that subcortical volume estimates are relatively robust across software versions [63]. Importantly, all structural MRI data were preprocessed using the same software version and our main analyses were therefore unaffected by the version change which was merely introduced to perform our exploratory follow-up analyses of subregions. Finally, our study design does not allow conclusions about directionality, as neuroimaging data, unlike maltreatment and externalizing problems, were assessed cross-sectionally. Longitudinal studies are required to draw causal inferences on the neural mechanisms linking childhood adversities to behavioral outcomes.

## Conclusion

Our findings underscore the importance of considering psychopathology to better capture the impact of child maltreatment on neural development – consistent with the ecophenotype model [27]. The model proposes that maltreated and non-maltreated individuals with the same psychiatric diagnosis exhibit distinct clinical and neurobiological features relevant for effective treatment. Although supported by research in patients with internalizing disorders [64], its applicability to externalizing psychopathology across development remains unresolved. Given the role of child maltreatment as a risk factor for CD and oppositional defiant disorder in general as well as specifically for the life-course persistent subtype [29, 30], individuals with maltreatment histories may represent a distinct subtype of patients with externalizing disorders displaying unique neurobiological features.

## Supplementary Information

Below is the link to the electronic supplementary material.

Supplementary Material 1 (PDF 889 KB)

## Data Availability

The data that support the findings of this study are not publicly available due to ethical and legal restrictions. Access to the data is restricted in accordance with data protection regulations.

## Footnotes

1 No subcortical volume differences emerged between no and low maltreatment-exposed in either externalizing subgroup (*p*s ≥.322), whereas participants with high maltreatment exposure differed from those with low maltreatment exposure in the high externalizing subgroup (*p*s ≤ .008).

2 When combining low and high maltreatment-exposed participants, the maltreatment by externalizing problems interaction remained significant for total hippocampal volume (*F*(1, 89) = 6.91, *p* = .010) and was reduced to trend level for total amygdala volume (*F*(1, 89) = 3.90, *p* = .051).
