# Supplementary material for: Subcortical brain structures among maltreated youth with high and low externalizing problems
Source: Eur Child Adolesc Psychiatry. 2025 Dec 8;35(4):1287–97. doi: 10.1007/s00787-025-02927-y (PMC13219078; doi:10.1007/s00787-025-02927-y)
Supplement: Supplementary file 1 — Supplementary Material 1 (PDF 889 KB) [file 787_2025_2927_MOESM1_ESM.pdf]

# Subcortical Brain Structures Among Maltreated Youth With High and Low Externalizing Problems

Charlotte C. Schulz[1,2], Lara M. C. Puhlmann[2,3,4], Lisa Folkens[1], Kai von Klitzing[1], Lorenz Deserno[2,5,6], Pascal Vrtička[2,7*], Lars O. White[1,8*]

[1] University of Leipzig, Department of Child and Adolescent Psychiatry, Psychotherapy, and Psychosomatics, Leipzig, Germany

[2] Max Planck Institute for Human Cognitive and Brain Sciences, Leipzig, Germany

[3] Leibniz Institute for Resilience Research, Mainz, Germany

[4] Technische Universität Dresden, Faculty of Psychology, Clinical Psychology and Behavioral Neuroscience, Dresden, Germany

[5] University of Würzburg, Department of Child and Adolescent Psychiatry, Psychosomatics and Psychotherapy, Centre of Mental Health, Würzburg, Germany

[6] Technische Universität Dresden, Department of Psychiatry and Psychotherapy, Dresden, Germany

[7] University of Essex, Department of Psychology, Centre for Brain Science, Colchester, United Kingdom

[8] University of Bremen, Department of Clinical Child and Adolescent Psychology and Psychotherapy, Bremen, Germany

*these authors contributed equally / share last authorship

**Corresponding Author**

Charlotte C. Schulz, M.Sc. (https://orcid.org/0000-0002-1082-4877)

University of Leipzig, Department of Child and Adolescent Psychiatry, Psychotherapy, and Psychosomatics, Liebigstraße 20a, 04103 Leipzig, Germany

Email: Charlotte.Schulz@medizin.uni-leipzig.de

**Table of Contents**

Appendix A ..... 3
Maltreatment Characteristics ..... 3
Psychopathological Symptoms ..... 3
Covariates ..... 4
Fetal Alcohol Syndrome ..... 4
Intelligence ..... 5
Puberty Status ..... 5
Socioeconomic Status ..... 5
Procedure ..... 5
Power Calculation ..... 7
MRI Data Pre-Processing ..... 7
Manual Corrections ..... 7
Hippocampal Subfield and Amygdala Nuclei Definition ..... 8
Data Analyses ..... 8
Analyses of Sample Characteristics ..... 8
Analyses of Categorical Main and Interaction Effects ..... 9
Analyses of Dimensional Main and Interaction Effects ..... 11
Within-Group Analyses of Dimensional Maltreatment Variables ..... 12
Supplemental Analyses ..... 12
Appendix B ..... 14
Sample Characteristics ..... 14
Main Results without Covariate Adjustment ..... 20
Main Effects of Maltreatment Exposure, Internalizing and Externalizing Problems ..... 20
Interaction Effects of Maltreatment Exposure, Internalizing and Externalizing Problems ..... 20
Within-Group Effects of Maltreatment Dimensions ..... 20
Sensitivity Analyses Testing Lateralization ..... 21
Main Effects of Maltreatment Exposure ..... 21
Main Effects of Internalizing Problems ..... 22
Maltreatment by Externalizing Problems Interaction ..... 23
Dimensional Moderation Effect ..... 26
Within-Group Effects of Maltreatment Exposure Level ..... 28
Associations with Amygdalar and Hippocampal Subregion Volumes ..... 29
Main Effects of Maltreatment, Internalizing and Externalizing Problems ..... 29
Interaction Effects of Maltreatment Exposure with Internalizing and Externalizing Problems ..... 30

Within-Group Effects of Maltreatment Exposure Level ... 31
Associations with Cortical Gray and Cerebral White Matter Volumes ... 33
Main Effects of Maltreatment Exposure, Internalizing and Externalizing Problems ... 33
Interaction Effects of Maltreatment Exposure with Internalizing and Externalizing Problems ... 34
Within-Group Effects of Maltreatment Exposure Level ... 34

## Appendix A

### Maltreatment Characteristics

The Maltreatment Classification System (MCS; [1]) distinguishes six maltreatment subtypes (i.e., physical neglect including lack of supervision and failure to provide as well as moral-legal/educational neglect, physical abuse, sexual abuse, and emotional maltreatment). For the latter dimension, we further differentiated between emotional abuse and emotional neglect. Perpetrator, severity, and subtype were determined for each event per developmental period: infancy (birth-17 months), toddler-hood (18 months-2 years), preschool age (3-5 years), early school age (6-7 years), late school age (8-12 years), and adolescence (13+ years).

The interviewers were extensively trained in conducting caregiver interviews and in applying the MCS coding system. Furthermore, the research team received on-site training and supervision by one of the MCS authors. We recorded all interview sessions for subsequent coding and to provide regular feedback to interviewers on their interview and coding performance, thereby facilitating standardization and reliability.

To obtain aggregate factor scores for the extent of maltreatment as well as abuse and neglect exposure, we computed measurement models for each exposure type by estimating latent factors for overall maltreatment as well as for abuse (i.e., physical, sexual, and emotional abuse) and neglect (i.e., physical and emotional neglect as well as moral-legal/educational maltreatment) from their respective chronicities, severities, and numbers of subtypes. Covariation between error variances of respective dimensional indicators of each exposure type (chronicity, severity, and subtype number) was accounted for by the model.

### Psychopathological Symptoms

At Wave 1 (see study timeline in **Figure A1**), conduct problems and emotional symptoms were measured with the Strengths and Difficulties Questionnaire (SDQ; [2]) from three different sources (i.e., mother, father, and teacher). The SDQ is a well-validated and reliable 25-item questionnaire which assesses children's psychopathological symptoms (i.e., emotional symptoms, conduct problems, hyperactivity/inattention, peer relationships, and prosocial behavior) during the last 6 months on a three-point scale (e.g., [3]). The current study focuses on emotional symptoms and

conduct problems as indicators of internalizing and externalizing problems, respectively. Based on population-derived cut-offs, categorical variables indicate average (0), borderline (1), and clinically-relevant symptoms (2) [4].

At Wave 1 and 2, caregivers also reported on adolescents' psychopathological symptoms using the Child Behavior Checklist (CBCL; [5]). The questionnaire assesses behavioral and emotional problems of children and adolescents aged 4-18 for the past 6 months on a scale from not true (0) to sometimes/somewhat true (1), and very/often true (2). It distinguishes eight syndromes (i.e., social withdrawal, somatic complaints, anxiety/depression, social, thought and attention problems, as well as delinquent and aggressive behavior), which can be summarized into the internalizing (including social withdrawal, somatic complaints, and anxiety/depression) and externalizing (including delinquent and aggressive behavior) problem scales. Based on the representative German norm sample (*N*=2856; [6]), we determined t-values for the internalizing and externalizing problem scales and classified participants into three groups each indicating average (0), borderline (1), and clinically-relevant internalizing and externalizing problems (2), respectively. Factorial validity and reliability of the CBCL could be confirmed [5–7].

To determine the presence of borderline or clinically relevant internalizing and externalizing problems across both waves, we combined parent- and teacher-reported SDQ and CBCL data using the "or rule". Typically, studies apply the "or rule" when researchers presuppose a low level of informant agreement [8, 9] as is arguably the case for parent and teacher reports [10, 11] and especially in the presence of maltreatment [12, 13]. Combined indicators for internalizing and externalizing problems across waves were translated into binary variables indicating above- or below-threshold borderline or clinically relevant internalizing (*n*=61) and externalizing (*n*=31) problems based on parent and teacher reports at either wave.

**Covariates**

***Fetal Alcohol Syndrome***

All participants were checked for the fetal alcohol syndrome (FAS) facial phenotype employing the FAS Facial Photographic Analysis Software, Version 2.1.0 [14] to rule out any effect of FAS on brain structure. Utilizing three photos of each participant, FAS features were assessed by a

4-Digit Diagnostic Code for facial phenotype rank ranging from absent to severe (1 to 4; [15]). The FAS facial photographic screening tool performed highly accurately in a foster care population [16]. When maternal alcohol exposure during pregnancy is unknown, however, only severe FAS features (rank 4) have been linked to a sufficiently positive predictive value and specificity to diagnose FAS [17].

***Intelligence***

We assessed participants' IQ utilizing the processing speed (symbol search, cancellation) and working memory (digit span, arithmetic) subscales of the Wechsler Intelligence Scale for Children – Fourth Edition (WISC-IV; [18]) for participants aged 12-15 or the Wechsler Adult Intelligence Scale – Fourth Edition (WAIS-IV; [19]) for participants aged 16-17, respectively. The average of the two subscale IQ scores for the respective test was calculated.

***Puberty Status***

Participants' puberty status was determined by applying the Tanner Scales [20, 21]. This sex-specific, picture-based questionnaire entails three questions assessing the onset and current status of physical development of external primary (breast/scrotum) and secondary (pubic hair) sex characteristics on a five-point scale from 0 to 4 indicating the stage of development. We calculated the mean across the responses to the questions regarding the current status (1) of primary sex characteristics and (2) of secondary sex characteristics.

***Socioeconomic Status***

We operationalized participants' socioeconomic status (SES) by the educational level (i.e., highest school qualification) of the mother (or other primary caregiver).

**Procedure**

The Wave 2 of the AMIS project (see study timeline in **Figure A1**) entailed two testing sessions (approximately 3 hours each) conducted by trained testing staff. At the Child and Adolescent Psychiatry of the University Clinic in Leipzig (Germany), adolescents first took part in the WISC-IV or WAIS-IV assessment and the photo session for FAS evaluation. Meanwhile, the caregiver reported on maltreatment incidents in the Maternal Maltreatment Classification Interview (MMCI; [22]) and filled out questionnaires. Afterwards, adolescents participated in a 20-minute mock scanning session

at the Max Planck Institute for Human Cognitive and Brain Sciences (MPI CBS; Germany). This procedure aimed at reducing motion artifacts due to nervousness and anxiety during the actual MRI scan. To that end, we accustomed participants to the scanning procedure by explaining to them why to remove any metal items, showing them all devices, informing them about the different scanner noises, and the importance of remaining as still as possible during scanning. In the mock scanner, participants received behavioral feedback regarding their head motion (measured with a motion sensor attached to their forehead) while watching a 10-minute animal documentary (i.e., the movie briefly froze when participants moved). This procedure helped to ensure that participants remained as motionless as possible during the actual scan. Furthermore, we could screen out one participant with excessive movement during the mock scan.

The second appointment took place at the MPI CBS. First, we instructed participants about the scanning procedure outside the scanner (including information on two functional MRI paradigms not further considered here). In the scanner, the structural scanning sequence was administered in-between the two functional paradigms. The participants spent approximately 60 minutes in the scanner. The adolescents completed questionnaires subsequent to the scanning procedure.

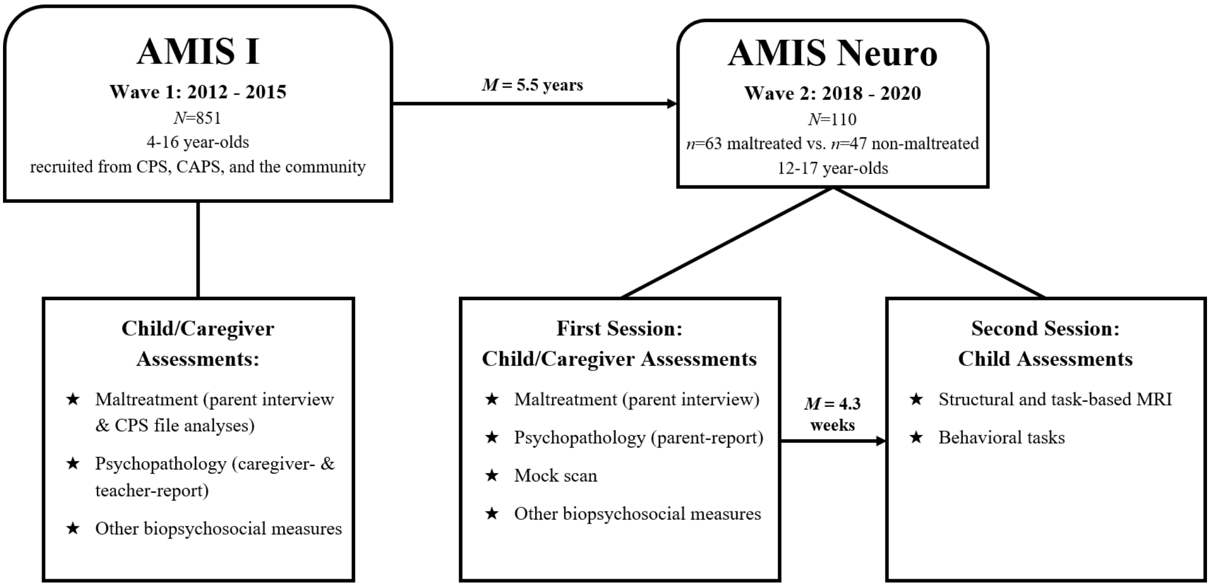


**Fig. A1** Study timeline of the research projects Analyzing Pathways from Childhood Maltreatment to Internalizing Symptoms and Disorders (AMIS) I and AMIS Neuro. Note: CPS=child protection services, CAPS=child and adolescent psychiatric services, MRI=magnetic resonance imaging

## Power Calculation

Before data acquisition, we conducted power calculations with G*Power, version 3.1.9.2 [23, 24] to determine the optimal sample size to investigate our hypothesized main effect of maltreatment. To this end, we focused on child and adolescent work showing overall effects of maltreatment on hippocampal volume, among others, as only preliminary findings regarding the effects of maltreatment dimensions were available at that time (i.e., 2016). Previous research findings on the general effects of maltreatment on neuroanatomy [25–29] suggested medium to large effect sizes ($d$=0.7 and $f$=0.35 according to [30]). When adjusting alpha for multiple comparisons ($\alpha$= 0.05/4), a sample size of $N$=120 was required to test the main effect of maltreatment with a desired power of .90 (F-test, 2 groups, $f$=0.35).

## MRI Data Pre-Processing

### *Manual Corrections*

To counter errors in gray (GM) and white matter (WM) surface placement introduced by the automatic preprocessing pipeline of Freesurfer, we conducted manual corrections to the GM and WM borders of each brain if necessary. The corrections were performed by two graduate students following a training period of 3 months, which aimed at ensuring reliability. As part of the training, the graduate students were instructed on identifying errors in pial surface and WM boundary placements. They further practiced to correct these errors by inserting control points to extend WM boundaries and appropriate deletions to allow for correction of pial surface placement. These corrections should prevent over- and underestimation of cortical thickness resulting from algorithm errors in surface placement. For each brain, corrections took approximately 3 to 4 hours. To maximize standardization and interrater reliability, both graduate students manually corrected 28 practice brains, which were compared weekly. Controlling for correction sequence effects, we randomly assigned experimental and control brains to the graduate students for corrections. Graduate students were blind to the respective maltreatment status during corrections. The trainer supervised them during the whole procedure.

***Hippocampal Subfield and Amygdala Nuclei Definition***

For each hemisphere, 9 amygdalar nuclei and 19 hippocampal subfields can be distinguished and extracted based on Freesurfer's automated segmentation pipeline [31, 32]. In line with previous research in the field [33, 34], we reduced the number of comparisons by merging amygdalar nuclei into two amygdalar segments, the basolateral (BLA) and centrocorticomedial (CMA) complex, based on functional aspects [35, 36]. Similarly, we focused on four subfields of the hippocampal formation, namely the cornu ammonis (CA) subfields 1, CA2/3, CA4/dentate gyrus (DG), and the subiculum, which have also been the focus of previous research related to childhood adversity [34, 37]. To this end, volume estimates for hippocampal subfields' head and body divisions were added for the subiculum, CA1, CA3 (including CA2), and CA4/DG, separately for each hemisphere. The latter also included head and body division for the granular cells and molecular layer of the DG (GC-ML-DG). Additionally, the indices for the BLA and CMA of the amygdala were calculated by summing up volume estimates for basal, lateral, accessory, paralaminar, corticoamygdalar, and anterior amygdaloid area nuclei as well as central, medial, and cortical nuclei, respectively, for each hemisphere. In a last step, means were calculated for each hippocampal subfield/amygdalar segment from the two volume estimates for each hemisphere. Thus, we examined the following: (a) four hippocampal subfields including the subiculum, CA1, CA3 (including CA2), CA4/DG as well as (b) two amygdalar segments including BLA and CMA. To examine hemispheric variation of subcortical volume estimates, we assessed Pearson's correlation coefficients between volume estimates for right and left hemispheres of the amygdala (see **Table B2** in **Appendix B**) as well as the hippocampus (see **Table B3** in **Appendix B**) and their respective subregions.

**Data Analyses**

All region-of-interest (ROI) analyses were conducted in IBM SPSS Statistics for Windows, v29.

***Analyses of Sample Characteristics***

The non-maltreated, low and high maltreatment-exposed groups were compared on relevant sample characteristics across all participants using one-way ANOVAs, Kruskal-Wallis, and Chi-squared ($\chi 2$) tests. For low and high maltreatment-exposed groups, we further assessed means and

standard deviations of the maltreatment dimensions and tested these for differences employing one-way ANOVAs.

To account for development-related and sex-specific differences in brain structure, we controlled all effects for age and gender using ANCOVAs or (moderated) multiple regression analyses. Correspondingly, we included intracranial volume (ICV) and SES as covariates in our analyses to control for the potentially confounding effects of head size (only in analyses of subcortical ROIs) and social environment (e.g., exposure to neighborhood violence or cognitive stimulation). Maltreatment effects were further controlled for recency and onset of the respective maltreatment exposure type (i.e. overall maltreatment, abuse, or neglect). In analyses assessing effects of mental health, we also included the respective other psychopathology type (i.e., externalizing or internalizing problems) utilizing ANCOVAs or (moderated) multiple regression analyses.

***Analyses of Categorical Main and Interaction Effects***

First, we tested main effects of (1) maltreatment exposure level (i.e., non-maltreated vs. low vs. high maltreatment-exposed participants) and (2) internalizing and externalizing problems (i.e., high vs. low externalizing and internalizing problems) regarding amygdalar and hippocampal volumes using one-way ANOVAs[1]. Secondly, we tested the interaction effects of maltreatment exposure with either externalizing or internalizing problems regarding amygdalar and hippocampal volumes applying two-way ANOVAs. Following up significant effects, we repeated the interaction analysis with a re-grouped binary maltreatment variable (i.e., combined no and low vs. high exposure level) in order to increase power and facilitate interpretation of the results.

Analyses were controlled for multiple comparisons (i.e., the number of ROIs). Thus, cut-off *p*-values were adjusted to $q<.025$. Following up significant main and interaction effects, one-way and two-way ANOVAs were employed to test lateralization of effects. Refer to the section **Sensitivity Analyses Testing Lateralization** in **Appendix B** for the results. These sensitivity analyses were

[1] Data were checked for outliers (i.e., residuals ± 3*interquartile range from the mean). One extreme outlier was detected in the supplemental subregion analyses and subsequently excluded for these analyses. Reported results for amygdalar and hippocampal subregions are thus based on effects without this outlier ($N$=97; 51.5% girls; age 14.74 ± 1.96 years). Matching of non-maltreated ($n$=40) vs. low ($n$=29) vs. high maltreatment-exposed ($n$=28) participants was preserved.

corrected for the number of comparisons (i.e., two hemispheres) adjusting cut-off *p*-values to $q<.025$. If the assumption of variance homogeneity was not met in any one-way ANOVA, we tested the respective effect using Welch's F test. If the residuals of any one-way ANOVA assessing main effects of (1) maltreatment exposure level and (2) externalizing or internalizing problems were not normally distributed, we tested the respective effect using (1) the Kruskal Wallis test and (2) the Mann-Whitney *U* test, respectively.

Following up significant maltreatment effects from omnibus tests, we conducted pairwise-comparisons of (a) low/high maltreatment-exposed vs. non-maltreated participants as well as (b) low vs. high maltreatment-exposed participants employing Bonferroni correction. Similarly, pairwise-comparisons were tested following up significant interaction effects comparing the three maltreatment exposure subgroups within the psychopathology groups (i.e., low vs. high internalizing and externalizing problems, respectively). Moreover, post-hoc tests were conducted to test pairwise-comparisons of (a) combined non-maltreated and low-maltreatment-exposed vs. high maltreatment-exposed within the psychopathology groups, (b) combined non-maltreated and low maltreatment-exposed as well as (c) high maltreatment-exposed across psychopathology groups, and (d) combined non-maltreated and low maltreatment-exposed with low internalizing or externalizing problems vs. high maltreatment-exposed with high internalizing or externalizing problems, respectively.

We modelled categorical (1) main effects of (a) maltreatment and (b) psychopathology (i.e., externalizing and internalizing problems, respectively) as well as (2) interaction effects with covariate adjustment as followed:

(1a) $Y_{ij} = \mu + \alpha_i + \gamma_1 ICV_{ij} + \gamma_2 Age_{ij} + \gamma_3 Gender_{ij} + \gamma_4 SES_{ij}\ (+ \gamma_5 Recency/Onset_{ij}) + \varepsilon_{ij}$

with $Y_{ij}$: measurement of person j in group i, $\mu$: overall mean, $\alpha_i$: main effect of maltreatment, $\gamma_{1\text{-}5}$: effects of covariates, $\varepsilon_{ij}$: individual error term,

(1b) $Y_{ij} = \mu + \beta_i + \gamma_1 ICV_{ij} + \gamma_2 Age_{ij} + \gamma_3 Gender_{ij} + \gamma_4 SES_{ij} + \gamma_5 EXT/INT_{ij} + \varepsilon_{ij}$

with $Y_{ij}$: measurement of person j in group i, $\mu$: overall mean, $\beta_i$: main effect of psychopathology, $\gamma_{1\text{-}5}$: effects of covariates, $\varepsilon_{ij}$: individual error term) and

(2) $Y_{ijk} = \mu + \alpha_i + \beta_j + (\alpha*\beta)_{ij} + \gamma_1 ICV_{ijk} + \gamma_2 Age_{ijk} + \gamma_3 Gender_{ijk} + \gamma_4 SES_{ijk} + \gamma_5 EXT/INT_{ijk}\ (+ \gamma_6 Recency/Onset_{ijk}) + \varepsilon_{ijk}$

with $Y_{ijk}$: measurement of person k in group defined by factors i and j, μ: overall mean, $\alpha_i$: main effect of maltreatment, $\beta_j$: main effect of psychopathology, $(\alpha*\beta)_{ij}$: interaction effect between maltreatment and psychopathology, $\gamma_{1-6}$: effects of covariates, $\varepsilon_{ijk}$: individual error term. We conducted analyses with and without adjustment for maltreatment recency and onset, respectively.

***Analyses of Dimensional Main and Interaction Effects***

We tested dimensional associations of concurrent parent-reported internalizing and externalizing problems with amygdala and hippocampus volumes using multiple regressions. To that end, we regressed subcortical ROI volumes on concurrent parent-reported internalizing and externalizing problems at Wave 2, respectively.

To follow-up significant categorical interaction effects, we conducted dimensional moderation analyses to examine whether concurrent parent-reported internalizing and externalizing problems at Wave 2 moderated the influence of maltreatment exposure level on amygdala and hippocampus volumes using Model 1 in the PROCESS macro, version 4.1 [38] in SPSS. Maximizing power for these follow-up analyses, we grouped no and low maltreatment-exposure groups together as described in the previous section to create a binary predictor. In order to include the moderation effect into the models, the respective dimensional predictors were mean-centered, their product was calculated, and tested for significance.

Following up significant dimensional effects, we conducted (moderated) multiple regression analyses to assess lateralization of the respective effects. As described in the previous section, the cut-off *p*-values for these sensitivity analyses were adjusted to $q<.025$.

We modelled dimensional (1) main effects of psychopathology as well as (2) moderation effects as followed (with covariate adjustment):

(1) $Y_j = \beta_0 + \beta_1 PSY_j + \gamma_1 ICV_j + \gamma_2 Age_j + \gamma_3 Gender_j + \gamma_4 SES_j + \gamma_5 EXT/INT_j + \varepsilon_j$

(2) $Y_j = \beta_0 + \beta_1 PSY_j + \beta_2 MAL_j + \beta_3 (MAL_j * PSY_j) + \gamma_1 ICV_j + \gamma_2 Age_j + \gamma_3 Gender_j + \gamma_4 SES_j + \gamma_5 EXT/INT_j (+ \gamma_6 Recency/Onset_j) + \varepsilon_j$

with $Y_j$: measurement of person j, $\beta_0$: intercept, $\beta_1 PSY_j$: effect of psychopathology (i.e., externalizing and internalizing problems, respectively) for person j, $\beta_2 MAL_j$: effect of maltreatment for person j (dummy coded: 0= no/low and 1=high maltreatment-exposed), $\beta_3(MAL_j*PSY_j)$: maltreatment by

psychopathology interaction effect for person j, $\gamma_{1\text{-}6}$: effects of covariates, $\varepsilon_j$: individual error term. We conducted analyses with and without adjustment for maltreatment recency and onset, respectively.

***Within-Group Analyses of Dimensional Maltreatment Variables***

We examined dose-dependent effects of dimensional maltreatment exposures (continuous aggregate scores for overall maltreatment as well as abuse and neglect using factor values from structural equation modeling) and volume estimates of the amygdala and hippocampus applying multiple regressions. To correct for multiple comparisons, we utilized the same corrected *p*-values as for the between-group analyses described in the section **Analyses of Categorical Main and Interaction Effects**. In the first step, we regressed subcortical ROI volumes on aggregate factor scores for overall maltreatment within maltreatment-exposed participants (*n*=58). In a second step, multiple regressions were conducted for each maltreatment exposure dimension (i.e., abuse and neglect) predicting subcortical ROI volumes separately within each of the exposure subgroups (either abused or neglected participants; *n*=47 each). Following up on significant effects of one exposure dimension, we included the respective other exposure dimension in the model. For all significant multiple regression analyses, if residuals were not normally distributed, bootstrapping was conducted (5000 bootstrapped samples drawn with replacement from the original dataset providing bias-corrected and accelerated (BCa) 95% CIs).

We modelled associations of (a) overall maltreatment, (b) abuse and neglect exposure with subcortical ROIs as followed (with covariate adjustment):

(a) $Y_j = \beta_0 + \beta_1 MAL_j + \gamma_1 ICV_j + \gamma_2 Age_j + \gamma_3 Gender_j + \gamma_4 SES_j\ (+ \gamma_5 Recency/Onset_j) + \varepsilon_j$

(b) $Y_j = \beta_0 + \beta_1 ABU_j + \beta_2 NEG_j + \gamma_1 ICV_j + \gamma_2 Age_j + \gamma_3 Gender_j + \gamma_4 SES_j\ (+ \gamma_5 Recency/Onset_j) + \varepsilon_j$

with $Y_j$: measurement of person j, $\beta_0$: intercept, $\beta_x MAL/ABU/NEG_j$: effect of maltreatment/abuse/ neglect exposure level for person j, $\gamma_{1\text{-}5}$: effects of covariates, $\varepsilon_j$: individual error term. We conducted analyses with and without adjustment for maltreatment recency and onset, respectively.

***Supplemental Analyses***

Mirroring the data-analytic approach for total hippocampal and amygdalar volumes described above, we tested for (1) categorical and (2) dimensional main and interaction effects across the whole

sample as well as (3) within-group effects of the dimensional maltreatment variables on volume estimates for amygdalar and hippocampal subregions as well as for global cortical GM and cerebral WM using (1) one-way and two-way ANOVAs, (2) (moderated) multiple regression analyses, as well as (3) multiple regression analyses. In line with the approach for the main analyses, we adjusted all analyses for covariates. As described in section **Analyses of Categorical Main and Interaction Effects**, Bonferroni corrected pairwise post-hoc comparisons were tested for significant categorical main and interaction effects. All analyses were controlled for multiple comparisons (i.e., the number of ROIs for (1) amygdalar segment, (2) hippocampal subfield, and (3) global brain volume estimates). Thus, cut-off *p*-values were adjusted to (1) $q<.025$, (2) $q<.0125$, (3) $q<.025$, respectively.

## Appendix B

### Sample Characteristics

In preliminary analyses, we examined whether non-maltreated, low and high maltreatment-exposed participants differed in important sample characteristics. There were no significant between-group differences in age, gender, handedness, IQ score, puberty status (i.e., the mean Tanner score), as well as FAS features (all *p*s>.05; see **Table B1**). However, the SES score differed between maltreatment-exposure groups ($H(2)=13.84$, $p<.001$). Thus, we controlled for this covariate in all between-group analyses comparing non-maltreated, low, and high maltreatment-exposed participants. Furthermore, as expected, a main effect of maltreatment-exposure group emerged for concurrent caregiver-reported internalizing ($F(2, 93)=6.76$ $p=.002$; $n=96$) and externalizing problems ($F(2, 95)=4.11$ $p=.019$). Post-hoc Bonferroni tests revealed that high maltreatment-exposed participants on average suffered from more internalizing and externalizing problems compared to non-maltreated participants (all *p*s<.05). No significant difference in internalizing and externalizing symptom level was found comparing the low maltreatment-exposed to the high maltreatment-exposed group (all *p*s≥.633). Similarly, low maltreatment-exposed did not significantly differ from the non-maltreated participants regarding externalizing symptom level ($p=.416$). However, the average internalizing symptom level was higher in participants with low maltreatment exposure than in the non-maltreated group ($p=.042$).

**Table B1**

*Sample and Maltreatment Characteristics*

| | Non-maltreated group (*n*=40) | Low maltreatment-exposed (*n*=29) | High maltreatment-exposed (*n*=29) | Between-group comparison | |
|---|---|---|---|---|---|
| **Sample characteristics** | | | | **Test statistic** | **p** |
| Mean age in years (*SD*) | 14.65 (1.89) | 14.66 (1.95) | 15.02 (2.13) | $F(2, 95)=0.35$ | .705 |
| % females | 60.00 | 41.38 | 48.28 | $\chi^2(2)=2.46$ | .293 |
| % left handedness | 12.50 | 10.35 | 10.35 | $\chi^2(2)=0.11$ | .946 |

| | | | | | |
|---|---|---|---|---|---|
| Median maternal school education | General qualification for university entrance | General qualification for university entrance | Secondary school certificate | *H*(2)=13.84 | <.001 |
| Mean IQ score (*SD*) | 99.08 (7.07) | 97.95 (9.77) | 95.45 (7.46) | *F*(2, 95)=1.72 | .184 |
| Mean Tanner score (*SD*) | 2.62 (0.90)[b] | 2.71 (0.97) | 2.59 (0.93) | *F*(2, 94)=0.14 | .873 |
| Mean FAS score (*SD*) | 1.20 (0.41) | 1.38 (0.49) | 1.48 (0.64)[c] | *F*(2, 52.30)=2.63 | .082[a] |
| Mean CBCL T2 internalizing problems (*SD*) | 50.08 (10.80) | 56.67 (11.48)[c] | 59.07 (9.34) | *F*(2, 93)=6.76 | .002 |
| Mean CBCL T2 externalizing problems (*SD*) | 48.08 (7.01) | 51.00 (8.95) | 53.66 (8.37) | *F*(2, 95)=4.11 | .019 |
| **Maltreatment characteristics (M, SD)** | | | | **Test statistic** | **p** |
| Chronicity[d] | | 38.85 (19.88) | 73.33 (24.77) | *F*(1, 56)=34.17 | <.001 |
| Max. severity[e] | | 2.00 (0.80) | 3.69 (0.93) | *F*(1, 56)=54.93 | <.001 |
| Number of subtypes[f] | | 1.28 (0.46) | 2.66 (0.86) | *F*(1, 56)=58.64 | <.001 |
| ***Abuse characteristics*** | | | | | |
| Chronicity[d] | | 9.10 (8.44) | 21.22 (14.40) | *F*(1, 45.22)=15.28 | <.001[a] |
| Max. severity[e] | | 1.31 (1.00) | 2.52 (1.43) | *F*(1, 56)=13.84 | <.001 |
| Number of subtypes[g] | | 0.79 (0.56) | 1.24 (0.79) | *F*(1, 56)=6.26 | .015 |
| ***Neglect characteristics*** | | | | | |
| Chronicity[d] | | 9.88 (9.98) | 22.60 (16.35) | *F*(1, 46.33)=12.78 | <.001[a] |
| Max. severity[e] | | 1.31 (1.23) | 3.17 (1.37) | *F*(1, 56)=29.84 | <.001 |
| Number of subtypes[g] | | 0.66 (0.48) | 1.72 (0.70) | *F*(1, 56)=45.61 | <.001 |

*Note.* FAS=Fetal Alcohol Syndrome, CBCL=Child Behavior Checklist, T2=Wave 2.

[a] If the assumption of variance homogeneity was not met, Welch's *F* test was used to examine differences between groups. [b] *n*=39. [c] *n*=27. [d] Chronicity=% of affected developmental periods; 1-100%. [e] 1-5. [f] 1-6. [g] 1-3.

**Table B2**

*Correlations between Volume Estimates for Hemispheres of the Amygdala and its Subregions*

| Variable | R AMY | L AMY | R BLA | L BLA | R CMA | L CMA |
|---|---|---|---|---|---|---|
| R AMY | ---- | .79*** | .90*** | .81*** | .66*** | .55*** |
| L AMY | | ---- | .77*** | .88*** | .60*** | .60*** |
| R BLA | | | ---- | .85*** | .73*** | .58*** |
| L BLA | | | | ---- | .62*** | .61*** |
| R CMA | | | | | ---- | .66*** |
| L CMA | | | | | | ---- |

*Note*. Pearson's product moment correlation coefficients were assessed within the total sample of $N$=97.

R=right, L=left, AMY=amygdala, BLA=basolateral complex, CMA=centrocorticomedial complex.

* $p < 0.05$. ** $p < 0.01$. *** $p < 0.001$.

**Table B3**

*Correlations between Volume Estimates for Hemispheres of the Hippocampus and its Subfields*

| Variable | R HC | L HC | R CA1 | L CA1 | R CA2/3 | L CA2/3 | R CA4/DG | L CA4/DG | R SUB | L SUB |
|---|---|---|---|---|---|---|---|---|---|---|
| R HC | ---- | .83*** | .85*** | .73*** | .61*** | .50*** | .81*** | .75*** | .70*** | .67*** |
| L HC | | ---- | .71*** | .87*** | .51*** | .69*** | .77*** | .88*** | .67*** | .64*** |
| R CA1 | | | ---- | .78*** | .66*** | .47*** | .81*** | .66*** | .58*** | .54*** |
| L CA1 | | | | ---- | .53*** | .75*** | .75*** | .87*** | .61*** | .51*** |
| R CA2/3 | | | | | ---- | .60*** | .83*** | .54*** | .31** | .27** |
| L CA2/3 | | | | | | ---- | .62*** | .81*** | .33*** | .18 |
| R CA4/DG | | | | | | | ---- | .78*** | .65*** | .58*** |
| L CA4/DG | | | | | | | | ---- | .66*** | .61*** |
| R SUB | | | | | | | | | ---- | .84*** |
| L SUB | | | | | | | | | | ---- |

*Note*. Pearson's product moment correlation coefficients were assessed within the total sample of $N$=97.

R=right, L=left, HC=hippocampus, CA=cornu ammonis, DG=dentate gyrus, SUB=subiculum.

* $p < 0.05$. ** $p < 0.01$. *** $p < 0.001$.

**Table B4**

*Correlations between Maltreatment and Mental Health Indicators*

| Variable | MAL Binary[a] | MAL Dim[b] | ABU[c] | NEG[c] | MAL Recency[b] | ABU Recency[c] | NEG Recency[d] | MAL Onset[b] | ABU Onset[c] | NEG Onset[d] | INT Cat[e] | EXT Cat[e] | INT T2[f] | EXT T2 |
|---|---|---|---|---|---|---|---|---|---|---|---|---|---|---|
| MAL Binary[a] | ---- | $.83^{***}$ | $.52^{***}$ | $.62^{***}$ | $.31^{*}$ | $.29^{*}$ | .28 | $-.55^{***}$ | $-.44^{**}$ | -.29 | $.23^{*}$ | $.23^{*}$ | $.26^{*}$ | $.24^{*}$ |
| MAL Dim[b] | | ---- | $.66^{***}$ | $.71^{***}$ | $.40^{**}$ | $.29^{*}$ | $.31^{*}$ | $-.59^{***}$ | $-.51^{***}$ | -.24 | .17 | .13 | .09[j] | .19 |
| ABU[c] | | | ---- | .14[g] | $.41^{**}$ | .28 | .15[h] | -.19 | $-.31^{*}$ | .27[h] | .06 | .14 | .09[i] | $.38^{**}$ |
| NEG[c] | | | | ---- | .21 | .14[g] | $.38^{**}$ | $-.52^{***}$ | -.23[g] | $-.56^{***}$ | -.01 | -.05 | .04 | -.13 |
| MAL Recency[b] | | | | | ---- | $.88^{***}$ | $.77^{***}$ | -.07 | .03 | .18 | -.12 | -.11 | -.08[j] | .04 |
| ABU Recency[c] | | | | | | ---- | $.48^{**}$[h] | -.10 | .02 | .22[h] | -.05 | -.12 | .14[i] | .26 |
| NEG Recency[d] | | | | | | | ---- | -.13 | .07[h] | -.06 | -.14 | -.25 | -.24[d] | -.17 |
| MAL Onset[b] | | | | | | | | ---- | $.77^{***}$ | $.77^{***}$ | $-.29^{*}$ | .01 | -.12[j] | -.16 |
| ABU Onset[c] | | | | | | | | | ---- | .30[h] | -.17 | -.09 | -.09[i] | $-.40^{**}$ |
| NEG Onset[d] | | | | | | | | | | ---- | -.14 | .19 | .02[d] | .23 |
| INT Cat[e] | | | | | | | | | | | ---- | $.30^{**}$ | $.54^{***}$ | $.35^{***}$ |
| EXT Cat[e] | | | | | | | | | | | | ---- | $.37^{***}$ | $.54^{***}$ |
| INT T2[f] | | | | | | | | | | | | | ---- | $.65^{***}$ |
| EXT T2 | | | | | | | | | | | | | | ---- |

*Note.* Pearson's product moment correlation coefficients were assessed within the total sample of *N*=98 (if not noted otherwise).

MAL=maltreatment, ABU=abuse, NEG=neglect, INT=internalizing symptoms, EXT=externalizing symptoms, T2=Wave 2.

[a] Coding maltreatment exposure: 0=no and low maltreatment exposure, 1=high maltreatment exposure. [b] *n*=58. [c] *n*=47. [d] *n*=46. [e] Coding mental health problems: low (0) vs. high (1) internalizing and externalizing problems, respectively. [f] *n*=96. [g] *n*=36. [h] *n*=35. [i] *n*=45. [j] *n*=56.

* $p < 0.05$. ** $p < 0.01$. *** $p < 0.001$.

**Table B5**

*Correlations between Brain Volume Estimates*

| Variable | ICV | GM | WM | AMY | BLA | CMA | HC | CA1 | CA2/3 | CA4/DG | SUB |
|---|---|---|---|---|---|---|---|---|---|---|---|
| ICV | ---- | .81*** | .87*** | .65*** | .66*** | .63*** | .70*** | .64*** | .55*** | .61*** | .54*** |
| GM | | ---- | .77*** | .62*** | .66*** | .56*** | .67*** | .62*** | .44*** | .59*** | .56*** |
| WM | | | ---- | .68*** | .70*** | .65*** | .67*** | .61*** | .46*** | .61*** | .53*** |
| AMY | | | | ---- | .92*** | .70*** | .68*** | .81*** | .51*** | .67*** | .49*** |
| BLA | | | | | ---- | .73*** | .76*** | .85*** | .56*** | .75*** | .56*** |
| CMA | | | | | | ---- | .65*** | .67*** | .51*** | .60*** | .46*** |
| HC | | | | | | | ---- | .88*** | .67*** | .89*** | .73*** |
| CA1 | | | | | | | | ---- | .71*** | .87*** | .61*** |
| CA2/3 | | | | | | | | | ---- | .83*** | .32** |
| CA4/DG | | | | | | | | | | ---- | .69*** |
| SUB | | | | | | | | | | | ---- |

*Note.* Pearson's product moment correlation coefficients were assessed within the total sample of $N$=98 or $N$=97 (for subregions).

ICV=intracranial volume, GM=cortical gray matter, WM=cerebral white matter, AMY=amygdala, BLA=basolateral complex, CMA=centrocorticomedial complex, HC=hippocampus, CA=cornu ammonis, DG=dentate gyrus, SUB=subiculum.

** $p < 0.01$. *** $p < 0.001$.

**Main Results without Covariate Adjustment**

***Main Effects of Maltreatment Exposure, Internalizing and Externalizing Problems***

There was no main effect of maltreatment exposure on bilateral hippocampal volume ($F$(2, 95)=1.10, $p$=.338). However, bilateral amygdala volume differed between the three exposure subgroups ($F$(2, 95)=5.69, $p$=.005, $\eta_p^2$=.11). Without covariate adjustment, no main effect of internalizing or externalizing problems emerged for the two subcortical ROIs (all $p$s≥.118). Similarly, dimensional indicators of concurrent internalizing and externalizing problems were not related to amygdalar and hippocampal volumes (all $p$s≥.389).

***Interaction Effects of Maltreatment Exposure, Internalizing and Externalizing Problems***

Maltreatment exposure interacted with externalizing problems to explain variance in bilateral amygdalar ($F$(2, 92)=6.89, $p$=.002, $\eta_p^2$=.13) and hippocampal volumes ($F$(2, 92)=6.38, $p$=.003, $\eta_p^2$=.12). In contrast, there were no interaction effects with internalizing problems for bilateral amygdalar and hippocampal volumes (all $p$s≥.356). Applying the re-grouped, binary maltreatment variable (i.e., no and low vs. high exposure level), the interaction effect between maltreatment exposure level and externalizing problems remained significant for amygdalar ($F$(1, 94)=16.26, $p$<.001, $\eta_p^2$=.15) and hippocampal volumes ($F$(1, 94)=13.72, $p$<.001, $\eta_p^2$=.13). Dimensional caregiver-reported externalizing problems at Wave 2 also moderated the effect of maltreatment exposure on amygdalar (β=-.40, $p$=.001, $R^2$=.16) and hippocampal volumes (β=-.32, $p$=.011, $R^2$=.08).

***Within-Group Effects of Maltreatment Dimensions***

Dimensional within-group analyses showed a negative dose-response relationship between the extent of overall maltreatment exposure and amygdala (β=-.41, $p$=.001, $R^2$=.17), but not hippocampus volume (β=-.19, $p$=.153). Within the subgroup of participants with a history of abuse ($n$=47), abuse exposure level was negatively related to total amygdala volume (β=-.48, $p$<.001, $R^2$=.23). This association remained significant when controlling for co-occurring neglect experiences (β=-.42, $p$=.004, $R^2$=.26). For the neglect dimension, however, no significant association with total amygdala volume emerged (β=-.11, $p$=.469; $n$=47). The dimensional indicators for the extent of abuse and neglect exposure were not significantly related to hippocampus volume (abuse: β=-.22, $p$=.143; neglect: β=.00, $p$=.997; $n$=47).

## Sensitivity Analyses Testing Lateralization

### *Main Effects of Maltreatment Exposure*

In exploratory analyses testing for lateralization, we found that the between-group effect of maltreatment exposure level was driven by volume differences in the left amygdala ($F(2, 91)=5.18$, $p=.007$, $\eta_p^2=.10$ with control of ICV, age, gender, and SES), but not in the right amygdala ($F(2, 91)=2.27$, $p=.109$ with control of all covariates). The between-group effect of maltreatment exposure level on left amygdala volume remained when including maltreatment recency ($F(2, 90)=5.17$, $p=.007$, $\eta_p^2=.10$) and onset ($F(2, 90)=4.24$, $p=.017$, $\eta_p^2=.09$) in the model. In line with the effects for total amygdala volume, Bonferroni-corrected post-hoc comparisons yielded significant mean left amygdala volume differences between low ($M=1861.83$, $SD=193.34$) vs. high maltreatment-exposed participants ($M=1681.11$, $SD=188.03$; $p=.002$). Moreover, there was a higher mean volume in low maltreatment-exposed compared to non-maltreated controls ($M=1731.36$, $SD=193.07$; $p=.019$). Further, the comparison of mean left amygdala volume between the non-maltreated and the high maltreatment-exposed group was non-significant ($p=.855$). Please refer to **Table B6** to view the results of the one-way ANCOVAs assessing main effects of maltreatment exposure level on bilateral, right and left amygdala volumes. In **Table B7**, Bonferroni-corrected post-hoc comparisons for the main effect of maltreatment exposure level on total amygdala volume are displayed.

**Table B6**

*Analyses of Covariance Assessing Between-Group Effects of Maltreatment on Amygdala Volume*

| Measure | Sum of Squares | df | Mean Square | *F* | *p* | $\eta_p^2$ |
|---|---|---|---|---|---|---|
| **Total amygdala volume (in mm$^3$)** | | | | | | |
| Intercept | 264908.23 | 1 | 264908.23 | 13.14 | <.001 | .13 |
| ICV (in cm$^3$) | 729540.03 | 1 | 729540.03 | 36.19 | <.001 | .29 |
| Age | 8949.07 | 1 | 8949.07 | 0.44 | .507 | .01 |
| Gender | 82384.71 | 1 | 82384.71 | 4.09 | .046 | .04 |
| SES | 3352.17 | 1 | 3352.17 | 0.17 | .684 | .00 |
| Maltreatment[b] | 170741.92 | 2 | 85370.96 | 4.24 | .017 | .09 |
| Error | 1834244.67 | 91 | 20156.54 | | | |
| **Right amygdala volume (in mm$^3$)** | | | | | | |

| | | | | | | |
|---|---|---|---|---|---|---|
| Intercept | 174265.49 | 1 | 174265.49 | 7.03 | .009 | .07 |
| ICV (in cm3) | 944178.92 | 1 | 944178.92 | 38.08 | <.001 | .30 |
| Age | 4195.88 | 1 | 4195.88 | 0.17 | .682 | .00 |
| Gender | 52760.29 | 1 | 52760.29 | 2.13 | .148 | .02 |
| SES | 4049.14 | 1 | 4049.14 | 0.16 | .687 | .00 |
| Maltreatment[b] | 112530.28 | 2 | 56265.14 | 2.27 | .109 | .05 |
| Error | 2256045.73 | 91 | 24791.71 | | | |
| **Left amygdala volume (in mm$^3$)** | | | | | | |
| Intercept | 374462.66 | 1 | 374462.66 | 15.18 | <.001 | .14 |
| ICV (in cm$^3$) | 542540.70 | 1 | 542540.70 | 22.00 | <.001 | .20 |
| Age | 15481.18 | 1 | 15481.18 | 0.63 | .430 | .01 |
| Gender | 118582.89 | 1 | 118582.89 | 4.81 | .031 | .05 |
| SES | 2720.98 | 1 | 2720.98 | 0.11 | .741 | .00 |
| Maltreatment[b] | 255606.80 | 2 | 127803.40 | 5.18 | .007 | .10 |
| Error | 2244313.60 | 91 | 24662.79 | | | |

*Note.* Analyses of covariance were conducted in a sample of *N*=98.

ICV=estimated intracranial volume, SES=socioeconomic status.

[a] Coding gender: 0=female, 1=male. [b] Coding maltreatment exposure: 0=non-maltreated, 1=low maltreatment exposure, 2=high maltreatment exposure.

**Table B7**

*Post-Hoc Tests for Between-Group Effect of Maltreatment Exposure on Amygdala Volume*

| | **Non-maltreated group** (*n*=40) | **Low maltreatment-exposed** (*n*=29) | **High maltreatment-exposed** (*n*=29) | **Non vs. Low Mal** | **Non vs. High Mal** | **Low vs. High Mal** |
|---|---|---|---|---|---|---|
| | *M (SD)* | *M (SD)* | *M (SD)* | *p* | *p* | *p* |
| Amygdala volume in mm$^3$ | 1775.54 (187.28) | 1880.68 (176.48) | 1719.49 (191.43) | .067 | .655 | .004 |

*Note.* Bonferroni-corrected pairwise comparisons following up the main effect of maltreatment exposure group on total bilateral amygdala volume.

***Main Effects of Internalizing Problems***

Following up the significant main effect of internalizing problems on total bilateral hippocampus volume, a lateralized main effect for left hippocampus volume emerged ($F(1, 91)=7.78$,

$p$=.006, $\eta_p^2$=.08 with control of ICV, age, gender, SES, and externalizing problems) with reduced volume in the high ($M$=4032.75, $SD$=403.55) vs. low internalizing problems groups ($M$=4184.37, $SD$=343.83). For right hippocampus volume, there was a non-significant trend for a main effect ($F$(1, 91)=4.13, $p$=.045, $\eta_p^2$=.43 with control of ICV, age, gender, SES, and externalizing problems; $q$>.025). However, for both hemispheres, there were no main effects of internalizing problems in analyses without covariate adjustment (all $p$s≥.060) pointing to a suppression effect in covariate-adjusted analyses.

### *Maltreatment by Externalizing Problems Interaction*

Follow-up analyses testing lateralization effects revealed a significant interaction of maltreatment group with externalizing problems for right amygdala volume ($F$(2, 87)=6.85, $p$=.002, $\eta_p^2$=.14 with control of ICV, age, gender, SES, and internalizing problems), but not left amygdala volume ($F$(2, 87)=2.76, $p$=.069 with control of all covariates). In contrast, there were significant interaction effects for both right ($F$(2, 87)=4.66, $p$=.012, $\eta_p^2$=.10 with control of all covariates) and left hippocampus volume ($F$(2, 87)=7.12, $p$=.001, $\eta_p^2$=.14 with control of all covariates). The interaction effects for right amygdalar as well as right and left hippocampal volumes remained significant when additionally controlling for maltreatment recency and onset (all $p$s≤.013). Please refer to **Table B8** to view the results of the interaction between maltreatment (3-level variable) and externalizing problems for total bilateral amygdala and hippocampus volumes.

**Table B8**

*Analyses of Covariance Assessing 3-Group Maltreatment by Externalizing Problems Interactions*

| Measure | Sum of Squares | df | Mean Square | *F* | *p* | $\eta_p^2$ |
|---|---|---|---|---|---|---|
| **Total amygdala volume (in mm$^3$)** | | | | | | |
| Intercept | 359381.96 | 1 | 359381.96 | 19.45 | <.001 | .18 |
| ICV (in cm$^3$) | 520830.53 | 1 | 520830.53 | 28.19 | <.001 | .25 |
| Age | 13820.44 | 1 | 13820.44 | 0.75 | .389 | .01 |
| Gender | 106925.79 | 1 | 106925.79 | 5.79 | .018 | .06 |
| SES | 13145.73 | 1 | 13145.73 | 0.71 | .401 | .01 |
| Internalizing problems[b] | 23978.21 | 1 | 23978.21 | 1.30 | .258 | .02 |
| Maltreatment[c] | 209746.67 | 2 | 104873.34 | 5.68 | .005 | .12 |

| | | | | | | |
|---|---|---|---|---|---|---|
| Externalizing problems[b] | 56.57 | 1 | 56.57 | 0.00 | .956 | .00 |
| Maltreatment[c] x externalizing problems[b] | 206316.35 | 2 | 103158.18 | 5.58 | .005 | .11 |
| Error | 1607313.74 | 87 | 18474.87 | | | |
| **Total hippocampus volume (in mm$^3$)** | | | | | | |
| Intercept | 1935093.99 | 1 | 1935093.99 | 33.76 | <.001 | .28 |
| ICV (in cm$^3$) | 3178984.09 | 1 | 3178984.09 | 55.46 | <.001 | .39 |
| Age | 36643.56 | 1 | 36643.56 | 0.64 | .426 | .01 |
| Gender[a] | 109106.95 | 1 | 109106.95 | 1.90 | .171 | .02 |
| SES | 322974.19 | 1 | 322974.19 | 5.63 | .020 | .06 |
| Internalizing problems[b] | 647140.18 | 1 | 647140.18 | 11.29 | .001 | .12 |
| Maltreatment[c] | 143389.34 | 2 | 71694.67 | 1.25 | .291 | .03 |
| Externalizing problems[b] | 4849.61 | 1 | 4849.61 | 0.09 | .772 | .00 |
| Maltreatment[c] x externalizing problems[b] | 792259.26 | 2 | 396129.63 | 6.91 | .002 | .14 |
| Error | 4987180.08 | 87 | 57323.91 | | | |

*Note.* Analyses of covariance were conducted in a sample of $N$=98.

ICV=estimated intracranial volume, SES=socioeconomic status.

[a] Coding gender: 0=female, 1=male. [b] Coding mental health problems: low (0) vs. high (1) internalizing and externalizing problems, respectively. [c] Coding maltreatment exposure: 0=non-maltreated, 1=low maltreatment exposure, 2=high maltreatment exposure.

Testing lateralization effects for the interaction of externalizing problems with the re-grouped maltreatment variable (i.e., combined no and low vs. high exposure level), significant interaction effects emerged for both right ($F$(1, 89)=13.25, $p$<.001, $\eta_p^2$=.13 with control of all covariates) and left amygdala volume ($F$(1, 89)=6.78, $p$=.011, $\eta_p^2$=.07 with control of all covariates). Similarly, interaction effects were significant for right ($F$(1, 89)=8.75, $p$=.004, $\eta_p^2$=.09 with control of all covariates) and left hippocampus volume ($F$(1, 89)=12.90, $p$<.001, $\eta_p^2$=.13 with control of all covariates). All interaction effects survived controlling for recency and onset of maltreatment incidents (all $p$s≤.004). Please refer to **Table B9** to view the results of the interaction between maltreatment exposure (re-grouped, binary variable) and externalizing problems for total bilateral amygdala and hippocampus volumes as well as to **Table B10** for the group means, standard deviations, and Bonferroni-corrected pairwise comparisons.

**Table B9**

*Analyses of Covariance Assessing 2-Group Maltreatment by Externalizing Problems Interactions*

| Measure | Sum of Squares | df | Mean Square | *F* | *p* | $\eta_p^2$ |
|---|---|---|---|---|---|---|
| **Total amygdala volume (in mm³)** | | | | | | |
| Intercept | 338813.67 | 1 | 338813.67 | 18.22 | <.001 | .17 |
| ICV (in cm³) | 520623.80 | 1 | 520623.80 | 28.00 | <.001 | .24 |
| Age | 12250.34 | 1 | 12250.34 | 0.66 | .419 | .01 |
| Gender | 123099.54 | 1 | 123099.54 | 6.62 | .012 | .07 |
| SES | 9213.31 | 1 | 9213.31 | 0.50 | .483 | .01 |
| Internalizing problems[b] | 21434.01 | 1 | 21434.01 | 1.15 | .286 | .01 |
| Maltreatment[c] | 171833.23 | 1 | 171833.23 | 9.24 | .003 | .09 |
| Externalizing problems[b] | 20584.84 | 1 | 20584.84 | 1.11 | .296 | .01 |
| Maltreatment[c] x externalizing problems[b] | 225237.80 | 1 | 225237.80 | 12.11 | <.001 | .12 |
| Error | 1654847.84 | 89 | 18593.80 | | | |
| **Total hippocampus volume (in mm³)** | | | | | | |
| Intercept | 1871616.46 | 1 | 1871616.46 | 32.85 | <.001 | .27 |
| ICV (in cm³) | 3135305.30 | 1 | 3135305.30 | 55.03 | <.001 | .38 |
| Age | 18574.38 | 1 | 18574.38 | 0.326 | .569 | .00 |
| Gender[a] | 111326.14 | 1 | 111326.14 | 1.95 | .166 | .02 |
| SES | 287264.63 | 1 | 287264.63 | 5.04 | .027 | .05 |
| Internalizing problems[b] | 689966.69 | 1 | 689966.69 | 12.11 | <.001 | .12 |
| Maltreatment[c] | 98709.27 | 1 | 98709.27 | 1.73 | .191 | .02 |
| Externalizing problems[b] | 64397.97 | 1 | 64397.97 | 1.13 | .291 | .01 |
| Maltreatment[c] x externalizing problems[b] | 728783.36 | 1 | 728783.36 | 12.79 | <.001 | .13 |
| Error | 5070571.87 | 89 | 56972.72 | | | |

*Note.* Analyses of covariance were conducted in a sample of *N*=98.

ICV=estimated intracranial volume, SES=socioeconomic status.

[a] Coding gender: 0=female, 1=male. [b] Coding mental health problems: low (0) vs. high (1) internalizing and externalizing problems, respectively. [c] Coding maltreatment exposure: 0=no and low maltreatment exposure, 1=high maltreatment exposure.

**Table B10**

*Post-Hoc Tests for the Interaction Effects of Maltreatment by Externalizing Problems on Subcortical Volumes*

| | **EXT-** | | **EXT+** | | **Comparison of EXT within MAL** | |
|---|---|---|---|---|---|---|
| | **AMY** | **HC** | **AMY** | **HC** | **AMY** | **HC** |
| | *M* (*SD*, *n*) | *M* (*SD*, *n*) | *M* (*SD*, *n*) | *M* (*SD*, *n*) | | |
| **MAL-** | 1788.40 (185.50, 52) | 4139.39 (342.16, 52) | 1915.58 (170.20, 17) | 4348.28 (277.81, 17) | *p*=.070 | *p*=.196 |
| **MAL+** | 1818.18 (194.88, 15) | 4292.67 (407.40, 15) | 1613.76 (121.47, 14) | 3907.50 (356.69, 14) | *p*=.015 | *p*=.020 |
| **Comparison of MAL within EXT** | *p*=1.00 | *p*=.797 | *p*<.001 | *p*=.004 | **Mal-, Ext- vs. Mal+, Ext+** | |
| | | | | | *p*=.009 | *p*=.168 |

*Note.* Means, standard deviations, and *p*-values for Bonferroni-corrected pairwise comparisons are displayed for total bilateral amygdala (AMY) and hippocampus volumes (HC; in $mm^3$) per binary maltreatment exposure (i.e., no/low maltreatment, Mal-, vs. high maltreatment exposure, Mal+) by externalizing problems groups (i.e., low, Ext-, vs. high externalizing problems, Ext+).

### *Dimensional Moderation Effect*

In sensitivity analyses assessing lateralization, neither for right nor left amygdala volume the dimensional moderation effect between maltreatment exposure (re-grouped, binary variable) and concurrent externalizing problems at Wave 2 survived when controlling for ICV, age, gender, SES, and internalizing problems (right amygdala: β=-.22, *p*=.034; left amygdala: β=-.24, *p*=.028; *q*>.025). Please refer to **Table B11** to view the results of the moderation analyses for total bilateral amygdala and hippocampus volumes.

**Table B11**

*Moderated Multiple Regression Analyses: Maltreatment, Concurrent Externalizing Problems, & Subcortical Volumes*

| Variable | β | *b* | *SE b* | *95% CI* LL | *95% CI* UL | *p* |
|---|---|---|---|---|---|---|
| **Total amygdala volume (in mm$^3$)** | | | | | | |
| Intercept | | 795.70 | 239.23 | 320.21 | 1271.20 | .001 |
| ICV (in cm$^3$) | .48 | 0.67 | 0.13 | 0.42 | 0.92 | <.001 |
| Age | -.08 | -7.37 | 7.59 | -22.46 | 7.73 | .335 |
| Gender[a] | .20 | 77.86 | 34.83 | 8.63 | 147.08 | .028 |
| SES | -.01 | -1.24 | 14.08 | -29.23 | 26.75 | .930 |
| Internalizing problems (T2) | .09 | 1.50 | 1.83 | -2.14 | 5.13 | .416 |
| Maltreatment[b] | -.16 | -66.57 | 35.52 | -137.16 | 4.02 | .064 |
| Externalizing problems (T2) | .049 | 1.14 | 2.78 | -4.39 | 6.67 | .684 |
| Maltreatment[b] x externalizing problems (T2) | -.24 | -9.94 | 4.07 | -18.04 | -1.85 | .017 |
| **Total hippocampus volume (in mm$^3$)** | | | | | | |
| Intercept | | 2307.74 | 435.07 | 1443.00 | 3172.49 | <.001 |
| ICV (in cm$^3$) | .62 | 1.62 | 0.23 | 1.16 | 2.07 | <.001 |
| Age | -.05 | -9.50 | 13.81 | -36.95 | 17.95 | .493 |
| Gender[a] | .16 | 117.35 | 63.34 | -8.55 | 243.25 | .067 |
| SES | -.12 | -38.27 | 25.61 | -89.17 | 12.62 | .139 |
| Internalizing problems (T2) | -.21 | -6.89 | 3.33 | -13.50 | -0.28 | .041 |
| Maltreatment[b] | -.06 | -43.43 | 64.59 | -171.81 | 84.96 | .503 |
| Externalizing problems (T2) | .25 | 10.87 | 5.06 | 0.81 | 20.94 | .035 |
| Maltreatment[b] x externalizing problems (T2) | -.15 | -11.73 | 7.41 | -26.45 | 2.99 | .117 |

*Note.* Moderated multiple regression analyses were conducted in a sample of *N*=96.

ICV=estimated intracranial volume, SES=socioeconomic status, T2=Wave 2.

[a] Coding gender: 0=female, 1=male. [b] Coding maltreatment exposure: 0=no and low maltreatment exposure, 1=high maltreatment exposure.

***Within-Group Effects of Maltreatment Exposure Level***

Follow-up sensitivity analyses controlling for multiple comparisons (i.e., two hemispheres; $q<.025$) indicated that the dimensional within-group effect of overall maltreatment level was driven by differences in left amygdala volume (β=-.36, $p$=.005, $R^2$=.43 with control of ICV, age, gender, and SES) rather than in right amygdala volume (β=-.24, $p$=.034 with control of all covariates). The association of left amygdala volume with overall maltreatment level remained significant when additionally controlling for maltreatment recency (β=-.39, $p$=.007, $R^2$=.43) and onset (β=-.37, $p$=.017, $R^2$=.43). In contrast, only right but not left amygdala volume was significantly associated with abuse exposure level (right: β=-.40, $p$<.001, $R^2$=.60 with control of all covariates; left: β=-.24, $p$=.096). This association also survived controlling for neglect experiences (β=-.40, $p$=.002, $R^2$=.60) as well as recency (β=-.38, $p$=.003, $R^2$=.61) and onset (β=-.41, $p$=.002, $R^2$=.61) of abuse exposure. Please refer to **Table B12** to view the results of within-group associations between overall maltreatment/abuse exposure level and total bilateral amygdala volume.

**Table B12**

*Multiple Regression Analyses Assessing Dimensional Association of Overall Maltreatment/Abuse Level with Amygdala Volume*

| Variable | β | *b* | *SE b* | *95% CI* | | *p* |
|---|---|---|---|---|---|---|
| | | | | LL | UL | |
| **Overall maltreatment level and total amygdala volume (in mm$^3$)** | | | | | | |
| Intercept | | 1050.91 | 251.74 | 545.75 | 1556.07 | <.001 |
| ICV (in cm$^3$) | .51 | .67 | 0.16 | 0.35 | 0.99 | <.001 |
| Age | -.19 | -18.98 | 10.00 | -39.04 | 1.08 | .063 |
| Gender | .15 | 59.63 | 46.37 | -33.41 | 152.68 | .204 |
| SES | -.08 | -13.68 | 19.99 | -53.79 | 26.44 | .497 |
| Maltreatment | -.32 | -467.14 | 163.84 | -795.90 | -138.37 | .006 |
| **Abuse level and total amygdala volume (in mm$^3$)** | | | | | | |
| Intercept | | 1118.32 | 313.09 | 485.55 | 1751.10 | <.001 |
| ICV (in cm$^3$) | .54 | 0.74 | 0.20 | 0.35 | 1.14 | <.001 |
| Age | -.22 | -21.43 | 11.19 | -44.04 | 1.19 | .063 |
| Gender | .02 | 8.90 | 54.61 | -101.48 | 119.28 | .871 |
| SES | -.04 | -6.26 | 22.52 | -51.78 | 39.26 | .782 |

| | | | | | | |
|---|---|---|---|---|---|---|
| Neglect | -.06 | -86.90 | 199.50 | -490.12 | 316.31 | .665 |
| Abuse | -.31 | -777.03 | 313.02 | -1409.67 | -144.39 | .017 |

*Note.* Multiple regression analyses were conducted within maltreatment-exposed (*n*=58) and abuse-exposed participants (*n*=47), respectively.

ICV=estimated intracranial volume, SES=socioeconomic status.

[a] Coding gender: 0=female, 1=male.

**Associations with Amygdalar and Hippocampal Subregion Volumes**

***Main Effects of Maltreatment, Internalizing and Externalizing Problems***

In categorical between-group analyses assessing the main effect of maltreatment exposure, BLA volume differences between the non-maltreated, low, and high maltreatment-exposed groups emerged ($F(2, 94)=4.74$, $p=.011$, $\eta_p^2=.09$). However, this effect did not survive controlling for ICV, age, gender, and SES ($F(2, 90)=2.04$, $p=.136$). Furthermore, there was no main effect of maltreatment exposure level for CMA volume ($F(2, 90)=2.99$, $p=.056$ with adjustment for ICV, age, gender, and SES) as well as for hippocampal subfield volume estimates (all *p*s≥.113 with covariate adjustment). Neither the internalizing nor externalizing problems groups exerted main effects on amygdalar subregion volumes (all *p*s≥.453 with adjustment for ICV, age, gender, SES, and the respective other psychopathology indicator). Moreover, there was no main effect of externalizing problems for hippocampal subfield volumes (all *p*s≥.240). Similarly, internalizing problems did not exert a main effect on CA1, CA4/DG, and subiculum volumes (all *p*s≥.028 with covariate adjustment; p-values adjusted for four hippocampal subfields, $q<.0125$). In contrast, a main effect of internalizing problems emerged for the hippocampal CA3 subfield ($F(1, 90)=8.83$, $p=.004$, $\eta_p^2=.09$ with adjustment for ICV, age, gender, SES, and externalizing problems) pointing to reduced CA3 volume in participants with high (*M*=208.08, *SD* =27.29) vs. low internalizing problems (*M* =219.36, *SD* =20.85). However, analyses without covariate adjustment reported a non-significant trend for this main effect ($F(1, 95)=4.64$, $p=.034$, $\eta_p^2=.05$) pointing to a suppression effect. Dimensional indicators of concurrent internalizing and externalizing problems at Wave 2 were not related to either amygdalar (all *p*s≥.169

with covariate adjustment) or hippocampal subregion volumes (all *p*s≥.079 with covariate adjustment).

***Interaction Effects of Maltreatment Exposure with Internalizing and Externalizing Problems***

However, maltreatment exposure interacted with externalizing problems to explain variance in BLA ($F$(2, 91)=5.89, $p$=.004, $\eta_p^2$=.12). This interaction effect remained significant when accounting for ICV, age, gender, SES, and internalizing problems ($F$(2, 86)=6.35, $p$=.003, $\eta_p^2$=.13) as well as recency ($F$(2, 85)=6.31, $p$=.003, $\eta_p^2$=.13) and onset ($F$(2, 85)=6.51, $p$=.002, $\eta_p^2$=.13). In contrast, there was no maltreatment exposure by externalizing problems interaction effect for amygdalar CMA volume ($F$(2, 86)=0.95, $p$=.390 with adjustment for all covariates). Moreover, significant interaction effects between maltreatment exposure and externalizing problems emerged for hippocampal CA1 ($F$(2, 86)=9.62, $p$<.001, $\eta_p^2$=.18), CA3 ($F$(2, 86)=4.75, $p$=.011, $\eta_p^2$=.10), CA4/DG ($F$(2, 86)=11.39, $p$<.001, $\eta_p^2$=.21), and subiculum volumes ($F$(2, 86)=5.04, $p$=.009, $\eta_p^2$=.11) with adjustment for ICV, age, gender, SES, and internalizing problems. The effects remained significant when additionally controlling for maltreatment recency and onset (all *p*s≤.011). In analyses without covariate correction, however, there was only a non-significant trend for a maltreatment exposure by externalizing problems interaction effect for the CA3 subfield ($F$(2, 91)=4.48, $p$=.014, $\eta_p^2$=.09; $q$<.0125) pointing to a suppression effect. In contrast, neither for amygdalar subregion nor hippocampal subfield volumes significant interaction effects with internalizing problems emerged (all *p*s≥.186 with adjustment for ICV, age, gender, SES, and externalizing problems).

No group differences in amygdalar and hippocampal subregion volume estimates emerged when comparing adolescents with no maltreatment to those with low maltreatment exposure within the subgroups of participants with low and high externalizing problems (all *p*s≥.486). Thus, we maximized power by grouping no and low maltreatment groups together for subsequent analyses. Applying the re-grouped, binary maltreatment variable (i.e., combined no and low vs. high exposure level), the interaction effect between maltreatment exposure level and externalizing problems groups remained significant for amygdalar BLA (see **Figure B1a**) as well as hippocampal CA1, CA3, CA4/DG, and subiculum volumes before (BLA: $F$(1, 93)=13.60, $p$<.001, $\eta_p^2$=.13; CA1: $F$(1, 93)=19.26, $p$<.001, $\eta_p^2$=.17; CA3: $F$(1, 93)=8.86, $p$=.004, $\eta_p^2$=.09; CA4/DG: $F$(1, 93)=17.92, $p$<.001,

$\eta_p^2$=.16; Subiculum: $F$(1, 93)=11.12, $p$=.001, $\eta_p^2$=.11) and after covariate adjustment (BLA: $F$(1, 88)=12.87, $p$<.001, $\eta_p^2$=.13; CA1: $F$(1, 88)=20.38, $p$<.001, $\eta_p^2$=.19; CA3: $F$(1, 88)=8.86, $p$=.004, $\eta_p^2$=.09; CA4/DG: $F$(1, 88)=20.91, $p$<.001, $\eta_p^2$=.19; Subiculum: $F$(1, 88)=9.41, $p$=.003, $\eta_p^2$=.10). Furthermore, the interaction effects for amygdalar BLA as well as hippocampal CA1, CA3, CA4/DG, and subiculum volumes survived controlling for maltreatment recency and onset (all $p$s≤.004).

We additionally tested the maltreatment by externalizing problems interaction as a dimensional moderation effect employing concurrent parent-reported externalizing symptoms at Wave 2 as a dimensional moderator. To this end, we compared participants with no and low maltreatment exposure to those with high exposure levels. In line with the categorical interaction effect reported above, caregiver-reported externalizing problems at Wave 2 also moderated the effect of maltreatment exposure on amygdalar BLA volume (β=-.23, $p$=.022, $R^2$=.56 with adjustment for ICV, age, gender, SES, and internalizing problems). There was only a non-significant trend for the moderation effect on BLA volume when additionally controlling for maltreatment recency (β=-.22, $p$=.028, $R^2$=.56) and onset (β=-.22, $p$=.026, $R^2$=.57; $q$<.025). In addition, a moderation effect of concurrent externalizing problems emerged for hippocampal CA1 volume (β=-.30, $p$=.006, $R^2$=.49 with covariate adjustment). This moderation effect also survived controlling for maltreatment recency and onset (all $p$s≤.008). While there was no moderation effect for subiculum volume (β=-.17, $p$=.128, $R^2$ =.40 with covariate adjustment), non-significant trends emerged for CA3 (β=-.21, $p$=.077, $R^2$=.32 with covariate adjustment) and CA4/DG subfield volumes (β=-.23, $p$=.036, $R^2$=.44 with covariate adjustment).

***Within-Group Effects of Maltreatment Exposure Level***

Dimensional within-group analyses testing associations between maltreatment exposure level and amygdala subregion volume estimates revealed a negative relationship between the extent of overall maltreatment exposure and BLA volume (β=-.41, $p$=.001, $R^2$=.17; see **Figure B1b**). This effect survived controlling for ICV, age, gender, and SES (β=-.31, $p$=.008, $R^2$=.53) as well as maltreatment recency (β=-.34, $p$=.010, $R^2$=.53). However, the association was reduced to a non-significant trend when including maltreatment onset into the model (β=-.26, $p$=.055, $R^2$=.54). The association between overall maltreatment exposure level and amygdalar CMA volume was non-

significant (β=-.21, $p$=.078, $R^2$=.52 with covariate adjustment). Furthermore, hippocampal CA1 volume was related to overall maltreatment exposure at trend-level (β=-.31, $p$=.017, $R^2$=.43 with covariate adjustment; $q$>.0125). This association, however, was significant, when additionally controlling for maltreatment recency (β=-.37, $p$=.011, $R^2$=.44), but not when adjusted for onset (β=-.25, $p$=.101, $R^2$=.44). There were no significant associations between overall maltreatment exposure level and CA3, CA4/DG, and subiculum volumes (with covariate adjustment all $p$s≥.152).

Moreover, amygdalar BLA volume was also negatively related to the level of abuse exposure (β=-.46, $p$=.001, $R^2$=.22). This association survived controlling for ICV, age, gender, and SES (β=-.28, $p$=.021, $R^2$=.56). However, when additionally including neglect experiences as covariate in the model, there was only a non-significant trend for abuse experiences on amygdalar BLA volume (β=-.27, $p$=.034, $R^2$=.57 adjusted for number of comparisons, i.e. two; $q$<.025). Yet, the extent of neglect exposure did not significantly predict amygdala BLA volume within this model (β=-.06, $p$=.634). The association between BLA volume and abuse exposure level did not survive additionally controlling for abuse recency (β=-.27, $p$=.038) and onset (β=-.26, $p$=.050). Moreover, the level of abuse exposure was also related to CMA volume (β=-.37, $p$=.012, $R^2$=.13). However, this association did not remain when including ICV, age, gender and SES (β=-.21, $p$=.077, $R^2$=.56) as well as the neglect dimension into the model (β=-.19, $p$=.124, $R^2$=.56). Additionally, a non-significant trend for a negative association between CA1 volume and abuse exposure level emerged (β=-.36, $p$=.013, $R^2$=.13), which did not survive correction for multiple comparisons (i.e., four hippocampal subfields; $q$<.0125) and covariate adjustment (β=-.17, $p$=.204). CA3, CA4/DG, and subiculum volume estimates were not associated with abuse exposure level (with covariate adjustment all $p$s≥.175). For the neglect dimension, no significant association with amygdalar (all $p$s≥.625 with adjustment for ICV, age, gender, SES, and abuse) and hippocampal subregion ROIs (all $p$s≥.649 with covariate adjustment) emerged.

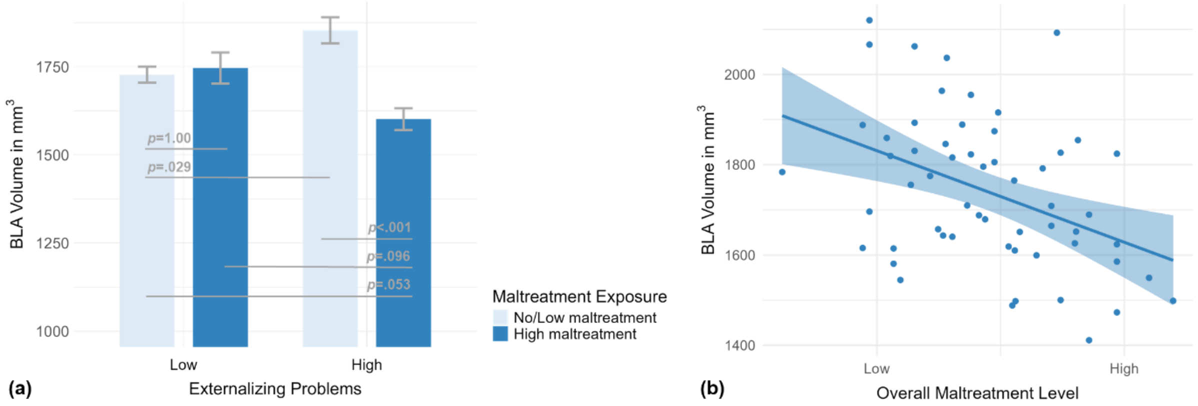


**Fig. B1** (a) Mean basolateral amygdala (BLA) volumes and respective standard errors for adolescents with combined no/low (light blue) vs. high maltreatment exposure (dark blue) within the subgroups of participants with low vs. high externalizing problems. (b) Negative association of overall maltreatment exposure level (x-axis) with BLA volume (y-axis) within participants exposed to maltreatment ($r$=-.41, $p$=.001; $n$=57)

## Associations with Cortical Gray and Cerebral White Matter Volumes

### *Main Effects of Maltreatment Exposure, Internalizing and Externalizing Problems*

In addition, we assessed main effects of maltreatment exposure on total cortical GM and cerebral WM volume (adjusting $p$-values for number of ROIs; $q$<.025). While no main effect of maltreatment emerged for cerebral WM volume ($F$(2, 92)=1.67, $p$=.195 with control of age, gender, and SES), the non-maltreated, low, and high maltreatment-exposed groups differed in mean cortical GM volume ($F$(2, 95)=4.26, $p$=.017, $\eta_p^2$=.08). This effect remained significant when including age, gender, and SES ($F$(2, 92)=4.73, $p$=.011, $\eta_p^2$=.09) as well as maltreatment recency ($F$(2, 91)=4.00, $p$=.022, $\eta_p^2$=.08) and onset ($F$(2, 91)=5.45, $p$=.006, $\eta_p^2$=.11) into the model. Bonferroni-corrected pairwise comparisons indicated a higher mean total cortical GM volume in low maltreatment-exposed ($M$=557275.80, $SD$=42189.49) compared to high maltreatment-exposed ($M$=522699.76, $SD$=47993.59; $p$=.013). However, low and high maltreatment exposed did not significantly differ from the non-maltreated group ($M$=540879.28, $SD$=45043.51) in respect to mean total cortical GM volume (all $p$s≥.306).

Furthermore, neither internalizing nor externalizing problems groups exerted main effects on the two ROIs (all *p*s≥.667 with control of age, gender, SES, and the respective other psychopathology dimension). Similarly, dimensional indicators of concurrent internalizing and externalizing symptoms at Wave 2 were not related to cerebral WM and cortical GM volume (all *p*s≥.434 with covariate adjustment).

***Interaction Effects of Maltreatment Exposure with Internalizing and Externalizing Problems***

We further tested the interaction of maltreatment exposure by externalizing and internalizing problems groups, respectively. Maltreatment exposure interacted with externalizing problems to explain variance in cerebral WM ($F(2, 88)=3.56$, $p=.033$, $\eta_p^2=.08$ with control of age, gender, SES, and internalizing problems) and cortical GM volume ($F(2, 88)=3.36$, $p=.039$, $\eta_p^2=.07$ adjusted for all covariates). The interaction effects, however, did not survive control for multiple comparisons ($q>.025$). Moreover, there was no interaction effect of maltreatment exposure with internalizing problems for cerebral WM and cortical GM volume (all *p*s≥.749 with control of age, gender, SES, and externalizing problems).

***Within-Group Effects of Maltreatment Exposure Level***

In dimensional within-group analyses, overall maltreatment level was negatively associated with total cortical GM volume (β=-.36, $p=.005$, $R^2=.13$; $n=58$ maltreated participants). This association remained significant when controlling for age, gender, and SES (β=-.35, $p=.004$, $R^2=.45$) as well as maltreatment recency (β=-.38, $p=.006$, $R^2=.45$). However, the association did not survive including maltreatment onset into the model (β=-.27, $p=.060$). In contrast, overall maltreatment level did not significantly relate to total cerebral WM volume (β=-.19, $p=.156$ adjusted for age, gender, and SES). Although abuse exposure level was related to total cerebral WM volume (β=-.35, $p=.017$, $R^2=.12$), this association did not survive controlling for neglect experiences as well as age, gender, and SES (β=-.13, $p=.411$). Total cortical GM volume was not significantly associated with abuse exposure level (β=-.04, $p=.789$ adjusted for age, gender, and SES, and neglect experiences). For neglect exposure level, no significant association with either total cortical GM or cerebral WM volume emerged (all *p*s≥.649).

**References**

1. Barnett D, Manly J, Cicchetti D (1993) Defining child maltreatment: The interface between policy and research. In: Cicchetti D, Toth SL (eds) Child abuse, child development, and social policy. Ablex, New York, pp 7–73
2. Goodman R (1997) The Strengths and Difficulties Questionnaire: a research note. J Child Psychol Psychiatry 38(5): 581–586. doi: 10.1111/j.1469-7610.1997.tb01545.x
3. Klein AM, Otto Y, Fuchs S et al. (2013) Psychometric Properties of the Parent-Rated SDQ in Preschoolers. Eur J Psychol Assess 29(2): 96–104. doi: 10.1027/1015-5759/a000129
4. Goodman R (2001) Psychometric properties of the strengths and difficulties questionnaire. J Am Acad Child Adolesc Psychiatry 40(11): 1337–1345. doi: 10.1097/00004583-200111000-00015
5. Achenbach TM (1991) Manual for the Child Behavior Checklist/4-18 and 1991 profile. Department of Psychiatry, University of Vermont, Burlington, VT
6. Arbeitsgruppe Deutsche Child Behavior Checklist (1998) Elternfragebogen über das Verhalten von Kindern und Jugendlichen; deutsche Bearbeitung der Child Behavior Checklist (CBCL/4-18). Einführung und Anleitung zur Handauswertung. 2. Auflage mit deutschen Normen, bearbeitet von M. Döpfner, J. Plück, S. Bölte, K. Lenz, P. Melchers & K. Heim. Arbeitsgruppe Kinder-, Jugend- und Familiendiagnostik (KJFD), Köln
7. Döpfner M, Schmeck K, Berner W et al. (1994) Zur Reliabilität und faktoriellen Validität der Child Behavior Checklist – eine Analyse in einer klinischen und einer Feldstichprobe [Reliability and factorial validity of the Child Behavior Checklist: An analysis of a clinical sample and a field sample]. Z Kinder Jugendpsychiatr Psychother 22: 189–205
8. Kendall PC, Hudson JL, Gosch E et al. (2008) Cognitive-behavioral therapy for anxiety disordered youth: a randomized clinical trial evaluating child and family modalities. Journal of consulting and clinical psychology 76(2): 282–297. doi: 10.1037/0022-006X.76.2.282
9. Goodman SH, Lahey BB, Fielding B et al. (1997) Representativeness of clinical samples of youths with mental disorders: a preliminary population-based study. Journal of abnormal psychology 106(1): 3–14. doi: 10.1037/0021-843X.106.1.3

10. Los Reyes A de, Augenstein TM, Wang M et al. (2015) The validity of the multi-informant approach to assessing child and adolescent mental health. Psychological bulletin 141(4): 858–900. doi: 10.1037/a0038498

11. Kraemer HC, Measelle JR, Ablow JC et al. (2003) A new approach to integrating data from multiple informants in psychiatric assessment and research: mixing and matching contexts and perspectives. The American journal of psychiatry 160(9): 1566–1577. doi: 10.1176/appi.ajp.160.9.1566

12. Lee JJ (2024) Cross-informant agreement between caregivers and teachers for prosocial behavior across child welfare settings. Children and youth services review 157. doi: 10.1016/j.childyouth.2024.107435

13. Romano E, Weegar K, Babchishin L et al. (2018) Cross-informant agreement on mental health outcomes in children with maltreatment histories: A systematic review. Psychology of Violence 8(1): 19–30. doi: 10.1037/vio0000086

14. Astley SJ (2016) FAS Facial Photographic Analysis Software. Fetal Alcohol Syndrome Diagnostic & Prevention Network, Seattle, WA

15. Astley SJ, Clarren SK (2000) Diagnosing the full spectrum of fetal alcohol-exposed individuals: introducing the 4-digit diagnostic code. Alcohol Alcohol 35(4): 400–410. doi: 10.1093/alcalc/35.4.400

16. Astley SJ, Stachowiak J, Clarren SK et al. (2002) Application of the fetal alcohol syndrome facial photographic screening tool in a foster care population. J Pediatr 141(5): 712–717. doi: 10.1067/mpd.2002.129030

17. Astley Hemingway SJ (2020) High facial specificity and positive predictive value are required to diagnose fetal alcohol syndrome when prenatal alcohol exposure is unknown. Adv Pediatr Res 7(4): 1-11. doi: 10.35248/2385-4529.20.7.44

18. Wechsler D (2003) Wechsler intelligence scale for children – fourth edition (WISC-IV). The Psychological Corporation, San Antonio, TX

19. Wechsler D (2008) Wechsler adult intelligence scale - fourth edition. Pearson Assessment, San Antonio, TX

20. Marshall WA, Tanner JM (1969) Variations in pattern of pubertal changes in girls. Arch Dis Child 44(235): 291–303. doi: 10.1136/adc.44.235.291
21. Marshall WA, Tanner JM (1970) Variations in the pattern of pubertal changes in boys. Arch Dis Child 45(239): 13–23. doi: 10.1136/adc.45.239.13
22. Cicchetti D, Toth SL, Manly JT (2003) Maternal maltreatment classification interview. Mt. Hope Family Center, University of Rochester, Rochester, New York
23. Erdfelder E, Faul F, Buchner A (1996) GPOWER: A general power analysis program. Behavior Research Methods, Instruments, & Computers 28(1): 1–11. doi: 10.3758/BF03203630
24. Faul F, Erdfelder E, Lang A-G et al. (2007) G*Power 3: a flexible statistical power analysis program for the social, behavioral, and biomedical sciences. Behavior research methods 39(2): 175–191. doi: 10.3758/BF03193146
25. Bauer PM, Hanson JL, Pierson RK et al. (2009) Cerebellar volume and cognitive functioning in children who experienced early deprivation. Biol Psychiatry 66(12): 1100–1106. doi: 10.1016/j.biopsych.2009.06.014
26. Carrion VG, Weems CF, Richert K et al. (2010) Decreased prefrontal cortical volume associated with increased bedtime cortisol in traumatized youth. Biol Psychiatry 68(5): 491–493. doi: 10.1016/j.biopsych.2010.05.010
27. Overfeld J, Nadig-Haynes K, Punder K de et al. (2016) Hippocampal volume in children aged 3–5 years with verified maltreatment exposure. Psychoneuroendocrinology 71: 40–41. doi: 10.1016/j.psyneuen.2016.07.107
28. Rao U, Chen L-A, Bidesi AS et al. (2010) Hippocampal changes associated with early-life adversity and vulnerability to depression. Biol Psychiatry 67(4): 357–364. doi: 10.1016/j.biopsych.2009.10.017
29. Teicher MH, Dumont NL, Ito Y et al. (2004) Childhood neglect is associated with reduced corpus callosum area. Biol Psychiatry 56(2): 80–85. doi: 10.1016/j.biopsych.2004.03.016
30. Cohen J (1992) A power primer. Psychol bull 112(1): 155-159

31. Iglesias JE, Augustinack JC, Nguyen K et al. (2015) A computational atlas of the hippocampal formation using ex vivo, ultra-high resolution MRI: Application to adaptive segmentation of in vivo MRI. Neuroimage 115: 117–137. doi: 10.1016/j.neuroimage.2015.04.042

32. Saygin ZM, Kliemann D, Iglesias JE et al. (2017) High-resolution magnetic resonance imaging reveals nuclei of the human amygdala: manual segmentation to automatic atlas. Neuroimage 155: 370–382. doi: 10.1016/j.neuroimage.2017.04.046

33. Oshri A, Gray JC, Owens MM et al. (2019) Adverse Childhood Experiences and Amygdalar Reduction: High-Resolution Segmentation Reveals Associations With Subnuclei and Psychiatric Outcomes. Child Maltreat 24(4): 400–410. doi: 10.1177/1077559519839491

34. Picci G, Christopher-Hayes NJ, Petro NM et al. (2022) Amygdala and hippocampal subregions mediate outcomes following trauma during typical development: Evidence from high-resolution structural MRI. Neurobiol Stress 18: 100456. doi: 10.1016/j.ynstr.2022.100456

35. LeDoux J (2007) The amygdala. Curr Biol 17(20): R868-R874

36. Solano-Castiella E, Anwander A, Lohmann G et al. (2010) Diffusion tensor imaging segments the human amygdala in vivo. Neuroimage 49(4): 2958–2965. doi: 10.1016/j.neuroimage.2009.11.027

37. Malhi GS, Das P, Outhred T et al. (2019) The effects of childhood trauma on adolescent hippocampal subfields. Aust N Z J Psychiatry 53(5): 447–457. doi: 10.1177/0004867418824021

38. Hayes AF (2018) Introduction to mediation, moderation, and conditional process analysis: A regression-based approach, 2nd ed. Guilford Press, New York, NY
